# Hypoxia-inducible factor 1 alpha limits dendritic cell stimulation of CD8 T cell immunity

**DOI:** 10.1371/journal.pone.0244366

**Published:** 2020-12-31

**Authors:** Charles W. Tran, Matthew J. Gold, Carlos Garcia-Batres, Kelly Tai, Alisha R. Elford, Megan E. Himmel, Andrew J. Elia, Pamela S. Ohashi

**Affiliations:** 1 Princess Margaret Cancer Centre, Toronto, Ontario, Canada; 2 Department of Immunology, University of Toronto, Toronto, Ontario, Canada; Duke University, UNITED STATES

## Abstract

Dendritic cells are sentinels of the immune system and represent a key cell in the activation of the adaptive immune response. Hypoxia-inducible factor 1 alpha (HIF-1α)–a crucial oxygen sensor stabilized during hypoxic conditions–has been shown to have both activating and inhibitory effects in immune cells in a context- and cell-dependent manner. Previous studies have demonstrated that in some immune cell types, HIF-1α serves a pro-inflammatory role. Genetic deletion of HIF-1α in macrophages has been reported to reduce their pro-inflammatory function. In contrast, loss of HIF-1α enhanced the pro-inflammatory activity of dendritic cells in a bacterial infection model. In this study, we aimed to further clarify the effects of HIF-1α in dendritic cells. Constitutive expression of HIF-1α resulted in diminished immunostimulatory capacity of dendritic cells *in vivo*, while conditional deletion of HIF-1α in dendritic cells enhanced their ability to induce a cytotoxic T cell response. HIF-1α-expressing dendritic cells demonstrated increased production of inhibitory mediators including IL-10, iNOS and VEGF, which correlated with their reduced capacity to drive effector CD8^+^ T cell function. Altogether, these data reveal that HIF-1α can promote the anti-inflammatory functions of dendritic cells and provides insight into dysfunctional immune responses in the context of HIF-1α activation.

## Introduction

Hypoxia inducible factor 1 (HIF-1) is a master regulator of the cellular response to hypoxia, and regulates multiple processes including angiogenesis, protein synthesis and glycolytic and oxidative metabolism [1]. HIF-1 belongs to a family of proteins that includes HIF-1α, HIF-1β (also known as aryl nucleotide receptor; ARNT) and HIF-2α, and functions as a heterodimeric transcription factor of HIF-1α and HIF-1β [2]. HIF-1β is constitutively expressed, while the expression of HIF-1α (and also HIF-2α) are tightly regulated. The regulation of HIF-1α is multi-faceted, with the most well-established pathway centred on the constitutive hydroxylation of HIF-1α on conserved, prolyl residues during normoxic conditions. HIF-1α hydroxylation by the PHD family of enzymes [3–6] facilitates its recognition by pVHL [7], which targets HIF-1α for degradation. During hypoxia, HIF-1α is no longer hydroxylated, and is able to translocate to the nucleus to dimerize with HIF-1β and carry out transcription of target genes. The cellular functions of HIF-1 in response to hypoxia have been well studied, particularly in the context of the tumor microenvironment. However, the characterization of its role in the immune system has been limited.

Several studies have shown that HIF-1α has important, but at times, opposing, effects on immune cell function. The loss of HIF-1α in macrophages and neutrophils reduces the severity of cutaneous inflammation and passive arthritis models [8], and HIF-1α deficient DCs were shown to have decreased expression of co-stimulatory markers compared to wildtype DCs and a reduced capacity to induce T cell proliferation [9]. HIF-1α has also been shown to be required for myeloid cell motility and bactericidal activity [10, 11]. Conversely, conditional deletion of HIF-1α in a subsequent study, using the same model as Cramer *et al*., resulted in exacerbated airway allergy in an allergic asthma model [12]. These data were attributed to the role of HIF-1α in promoting anti-inflammatory functions of interstitial macrophages, a population that was also affected when using the *LysM-Cre* conditional knockout model. HIF-1α upregulation has also been observed in macrophages following exposure to heat-killed bacteria in normoxic conditions [11], and several studies have shown that HIF-1α is stabilized downstream of TLR or LPS stimulation [13–16].

In dendritic cells (DCs), opposing functions for HIF-1α have also been reported by various groups. One study showed that DCs exposed to hypoxic conditions, increased the production of pro-inflammatory cytokines [17], while another study showed that the loss of HIF-1α in DCs unexpectedly resulted in increased pathology in a DSS-colitis model [18]. Additional studies have suggested that HIF-1α inhibits pro-inflammatory DC function and downstream induction of CD8^+^ T cell responses in vitro [19], and dampens dendritic cell function in a *Leishmania* infection model [20, 21].

HIF-1α has been shown to be a crucial mediator of glycolytic re-programming in pro-inflammatory DCs [16]. Metabolic re-programming of DCs towards glycolysis is suggested to be necessary for CCR7-dependent migration of DCs to draining lymph node, and CCR7 stimulation has also been found to further drive DC metabolic reprogramming towards glycolysis in a HIF-1α-dependent manner [22].

In our studies, we examined the consequences of either constitutive HIF-1α expression or HIF-1α deletion on DC function. For constitutive HIF-1α expression, we used a model where a form of HIF-1α that cannot be hydroxylated was expressed in DCs. This form of HIF-1α escapes recognition by pVHL and cannot be degraded through this pathway. These DCs were then tested *in vitro* and *in vivo* to investigate the functional outcome of sustained HIF-1α expression in DCs on the immune response.

## Results

### Constitutive expression of HIF-1α in dendritic cells does not alter the expression of activation or inhibitory markers

**Fig 1:**
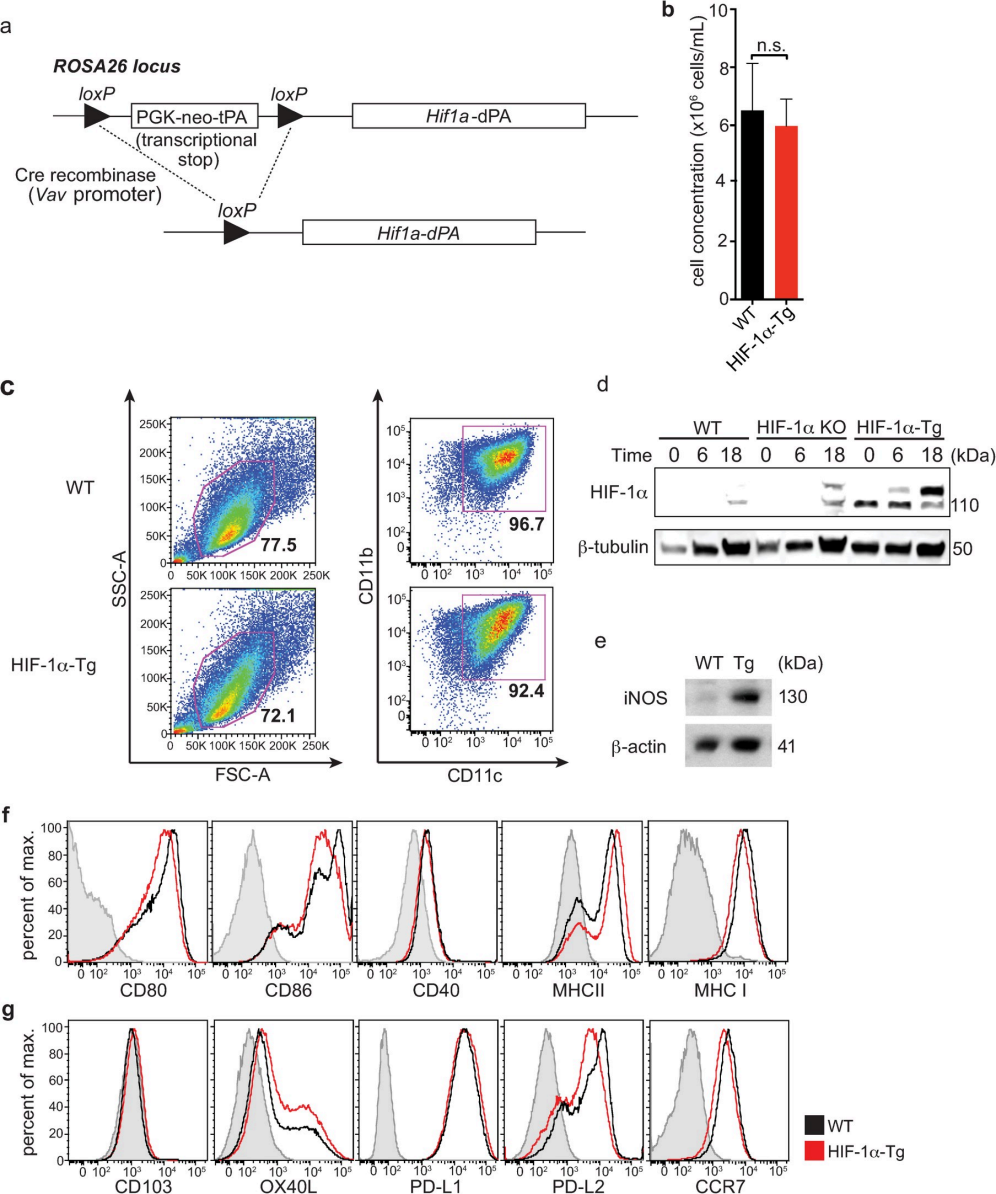
Constitutive expression of HIF-1α in BMDCs leads to increased iNOS expression without affecting surface levels of co-stimulatory or co-inhibitory molecules. (**a**) *Hif1*a-dPA loxP-stop-loxP mice express a transgene containing a floxed transcriptional stop cassette upstream of a mutated HIF-1**α** with two proline to alanine substitutions (P402A and P564A; HIF-1**α**-dPA). (**b**) Average cell concentration following 10 days of BM culture in the presence of GM-CSF (data shown is representative of 4 independent experiments). (**c**) BMDC frequencies, based on FSC/SSC and CD11c^+^CD11b^+^ profiles, are similar between WT and HIF-1**α**-Tg cultures. (**d**) Constitutive HIF-1**α** expression was confirmed in HIF-1**α**-Tg BMDCs by Western blot before or after stimulation with CpG for 16-20h. (**e**) Expression of iNOS (a direct HIF target) by Western blot in CpG-stimulated, WT and HIF-1**α**-Tg BMDCs. (**f**) BMDCs were left unstimulated or stimulated with CpG, and stained for various DC activation markers and (**g**) other surface molecules including CD103, OX40L, PD-L1, PD-L2, and CCR7. For all plots, the solid black line indicates WT BMDCs and the solid red line indicates HIF-1**α**-Tg BMDCs. FMO controls are indicated by the shaded grey histogram. For experiments with CpG stimulation of BMDC, stimulation was performed by adding CpG to BMDCs at a concentration of 10μM and incubating for 16-20h. Western blot data are representative of two independent experiments, and flow cytometry data are representative of at least five independent experiments. For (**b**), n.s.: not significant using Student’s *t*-test (two-tailed).

To study the consequences of HIF-1α expression in dendritic cell function, we obtained loxP-stop-loxP-*Hif1a*-dPA transgenic mice that possess a mutant form of HIF-1α with P402A and P564A substitutions, placed downstream of a floxed transcriptional stop cassette [23]. Mutation of these proline residues in HIF-1α abrogates their hydroxylation and subsequent recognition by pVHL, and therefore prevents the degradation of HIF-1α through this pathway. We crossed these loxP-stop-loxP *Hif1a*-dPA mice to *Vav-Cre* mice to generate loxP-stop-loxP-*Hif1a*-dPa *Vav-Cre*^+^ (herein referred to as HIF-1α-Tg) mice, enabling constitutive expression of HIF-1α in all hematopoietic cells (Fig 1A). We generated BMDC by culturing bone marrow from HIF-1α-Tg mice in the presence of GM-CSF and did not observe any differences between WT and HIF-1α-Tg in either CD11c^+^MHCII^+^ BMDC growth, frequency or viability (Fig 1B and 1C, S1A Fig). As expected, we confirmed increased expression of HIF-1α in HIF-1α-Tg BMDCs compared to WT (Fig 1D). Interestingly, levels of HIF-1α were further upregulated following CpG-stimulation of HIF-1α-Tg BMDCs. In *Hif1a*^*fl/fl*^ *Cd11c-Cre-Gfp* (HIF-1α KO) BMDCs, some HIF-1α signal was unexpectedly observed in HIF-1α KO BMDCs following 18h of CpG stimulation (Fig 1D). However, expression of HIF-1α target genes including *Ldha* and *Slc2a1* were significantly reduced in HIF-1α KO BMDCs (S2 Fig), as expected. Consistent with this data, we observed increased expression of several HIF-1α target genes including *Slc2a1* [24] and *Nos2* [25] in HIF-1α-Tg compared to WT BMDCs following CpG stimulation (S2 Fig). Expression of other genes including *Adora2b*, *Arg1* and *Epo* were also elevated, but not significantly increased in HIF-1α-Tg BMDCs following stimulation, while *Arg2* was not differentially altered in HIF-1α-Tg BMDCs. We further confirmed increased protein levels of iNOS in HIF-1α-Tg DCs (Fig 1E). Next, the surface expression of conventional activation markers on HIF-1α-Tg BMDCs was examined to determine whether any changes could be detected compared to WT BMDCs. No major differences in the levels of CD80, CD86, CD40, MHC II or MHC I were found between WT and HIF-1α-Tg BMDCs following stimulation with CpG (Fig 1F). The expression levels of other markers such as CD103, OX40L, CCR7 or inhibitory molecules including PD-L1 (a predicted HIF-1α target [26]) and PD-L2, were also found to be similar between WT and HIF-1α-Tg BMDCs (Fig 1G). Additionally, no differences in antigen uptake or processing were detected (S1B–S1D Fig). These data indicated that constitutive and functional HIF-1α expression in BMDCs was induced, and that sustained expression of HIF-1α did not alter the number of BMDCs nor their surface levels of activation or inhibitory markers.

### Enforced expression of HIF-1α in dendritic cells results in an impaired response in vivo

**Fig 2:**
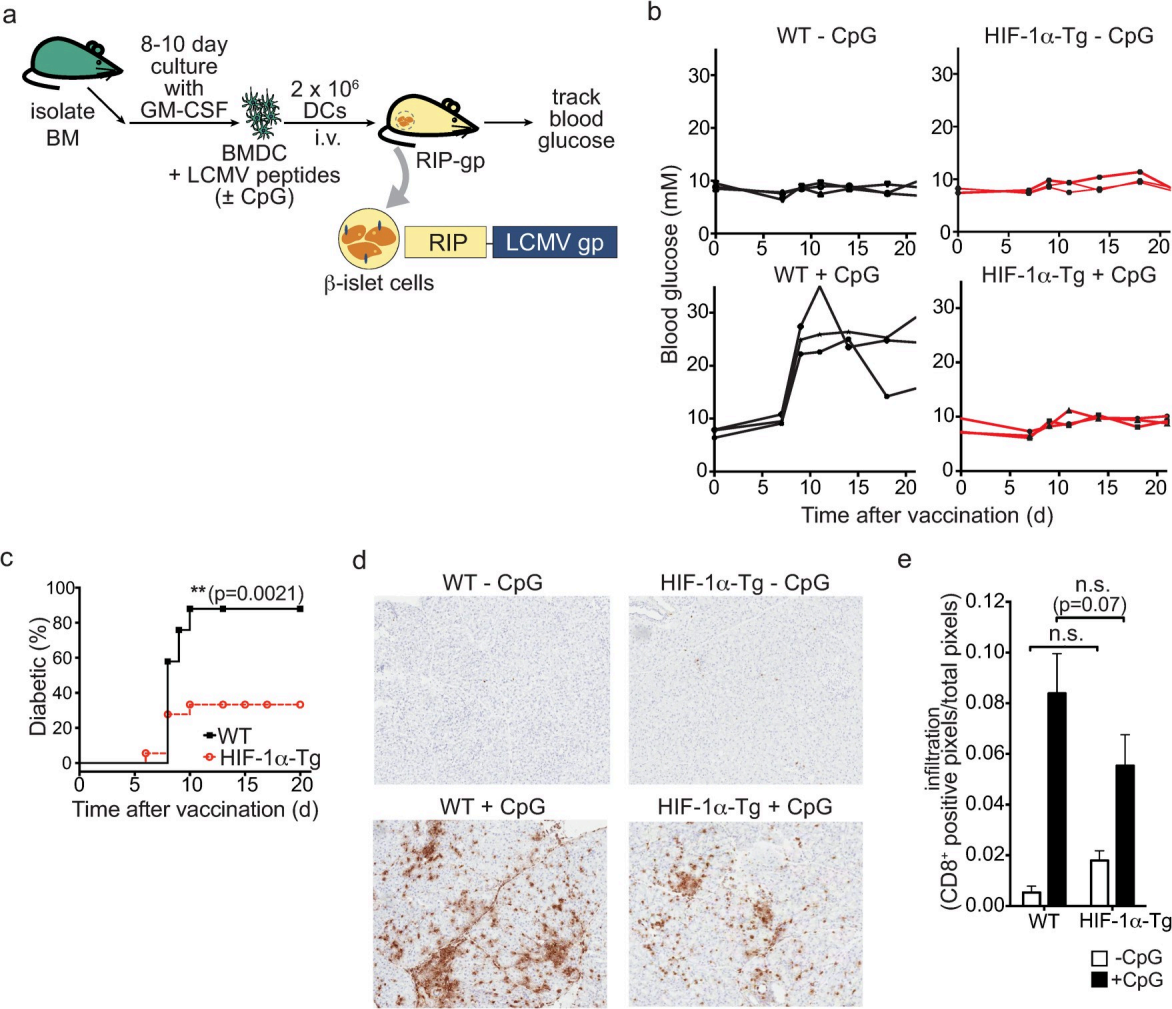
Overexpression of HIF-1α-dPA in BMDCs results in impaired induction of immune pathology. (**a**) Experimental overview of the RIP-gp BMDC vaccination model. BMDCs were harvested following 8 days of culture, stimulated with ODN 1826 CpG, pulsed with LCMV gp peptides, and given intravenously into RIP-gp mice. (**b**) Blood glucose measurements of RIP-gp mice receiving gp-peptide pulsed, unstimulated or CpG-stimulated, WT or HIF-1**α**-Tg BMDCs (*n* = 3 mice per group; data representative of three independent experiments). (**c**) Cumulative diabetes incidence in RIP-gp mice receiving B6 or HIF-1**α**-Tg, CpG-stimulated, gp peptide-pulsed BMDCs. Data are representative of 20–25 mice per group. (**d**) Representative CD8^+^ staining of pancreatic sections 6 days following BMDC administration (10X magnification). (**e**) Quantification of pancreatic CD8^+^ infiltration in RIP-gp mice. C57BL/6 or HIF-1**α**-Tg BMDCs were administered to RIP-gp mice and 6 days later, the pancreas was snap-frozen and processed for histology. CD8^+^ staining was quantified using positive pixel detection. *P ≤ 0.05; n.s.: not significant. Error bars represent S.D. For (**c**), a log-rank (Mantel-Cox) test was used to determine significance, and for (**e**), a two-way ANOVA was performed with Tukey’s test for multiple comparisons.

A series of experiments were performed to evaluate the immunostimulatory capacity of HIF-1α-Tg BMDCs *in vivo*. We have developed an assay to evaluate DC function *in vivo* using the RIP-gp model. In this transgenic model, the lymphocytic choriomeningitis virus glycoprotein (LCMV-gp) is expressed by the β-islet cells of the pancreas using the rat insulin promoter (RIP). Studies have shown that the T cells specific for the LCMV-gp remain ignorant or naïve and can be activated by a variety of stimuli [27]. We have shown that DCs can be generated from the bone marrow, pulsed with gp peptides, and matured with various TLR stimuli to induce an LCMV gp-specific cytotoxic response that leads to the destruction of β-islet cells and diabetes [28, 29]. Therefore, we examined whether HIF-1α-Tg DCs could stimulate a functional CD8^+^ T cell response in the RIP-gp model (Fig 2A). As expected, CpG-stimulated, WT BMDCs pulsed with LCMV peptides were able to induce diabetes in RIP-gp mice after approximately 8–10 days. In contrast, RIP-gp mice receiving CpG-matured HIF-1α-Tg BMDCs showed reduced incidence of diabetes compared to mice immunized with wildtype BMDCs (Fig 2B and 2C), while HIF-1α KO CpG-stimulated BMDCs had similar diabetes induction compared to WT (S3 Fig). Reduced CD8^+^ infiltration was observed in the pancreas from RIP-gp mice that were given HIF-1α-Tg BMDCs; however, this difference was not statistically significant (Fig 2E). Therefore, HIF-1α-Tg DCs have a limited capacity to induce a gp-specific response *in vivo* compared to wildtype DCs.

### Impaired induction of cytotoxic T cell responses with BMDCs constitutively expressing HIF-1α

**Fig 3:**
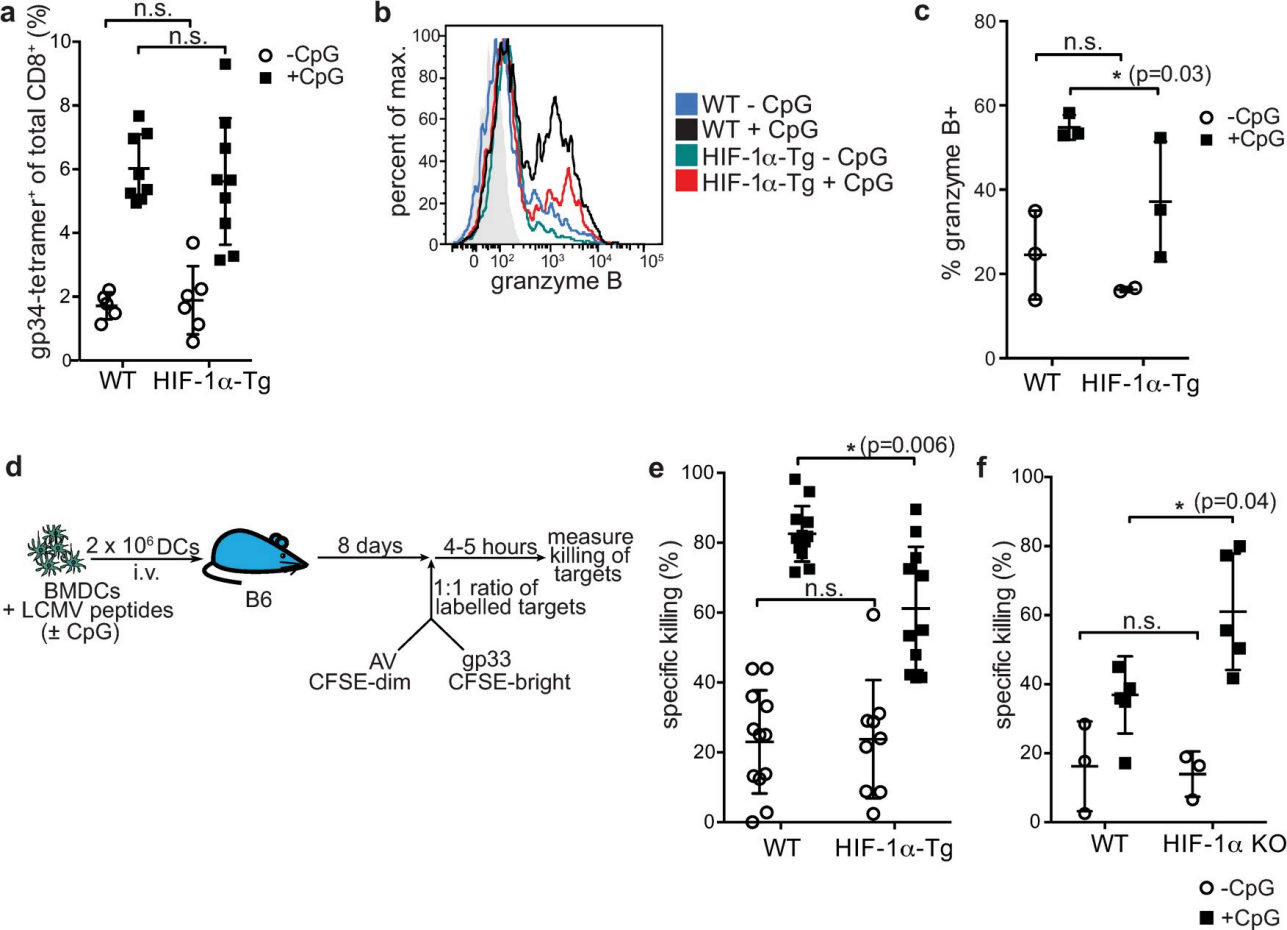
Constitutive expression of HIF-1α-dPA in BMDCs limits cytotoxic CD8^+^ T cell responses. (**a**) RIP-gp mice were vaccinated with gp-peptide pulsed, unstimulated or CpG-stimulated, WT or HIF-1**α**-Tg BMDCs. Eight days later, blood was collected for tetramer staining of gp-specific CD8^+^ T cells (gp34-tetramer^+^). (**b**) Pancreatic infiltrating lymphocytes (PIL) were isolated from RIP-gp mice 6 days following WT or HIF-1**α**-Tg BMDC vaccination and stained for granzyme B. Representative staining for CD8^+^ PIL is shown in the histogram and (**c**) the percentage of granzyme B^+^ PIL (of CD8^+^CD45^+^ cells) is shown. (**d**) Overview of the CTL assay used to assess antigen-specific killing of targets *in vivo*. Peptide-pulsed, CpG stimulated or unstimulated, WT or HIF-1**α**-Tg BMDCs were infused into naïve B6 host animals. Eight days later, fluorescently labeled, peptide-pulsed target cells were given i.v., and spleens of recipient mice were harvested and analyzed by flow cytometry to assess the antigen-specific, direct killing capacity of CTLs in vaccinated mice. CTL data for mice vaccinated with (**e**) WT or HIF-1**α**-Tg BMDCs or (**f**) WT or HIF-1**α** KO BMDCs, is shown. For (**e**) and (**f**), open circles represent mice given unstimulated BMDCs (-CpG), while shaded squares represent mice given CpG-stimulated BMDCs (+CpG). Data are representative of 2–3 independent experiments, with 2–3 mice per group. For (**a**) and (**e**), data were pooled from three independent experiments. *P ≤ 0.05; n.s.: not significant. Statistical testing was performed with two-way ANOVA and Tukey’s test.

Further experiments were done to examine the impact of constitutive HIF-1α expression in BMDCs on the induction of immune responses *in vivo*. We measured the expansion of gp34-tetramer-specific CD8^+^ T cells in the blood of RIP-gp mice by flow cytometry following WT and HIF-1α-Tg BMDC vaccination (Fig 3A). The frequency of CD8^+^ gp34-tetramer^+^ cells was slightly, but not significantly reduced in mice receiving HIF-1α-Tg BMDCs, while HIF-1α-KO BMDCs had similar expansion of gp34-tetramer-specific CD8^+^ T cells as WT (S3 Fig). To examine whether differences in the functional properties of CD8^+^ T cells could be found in the pancreas, pancreatic infiltrating lymphocytes (PIL) were isolated and stained for granzyme B expression. Notably, a reduced percentage of PIL expressed granzyme B when RIP-gp mice were given HIF-1α-Tg BMDCs (Fig 3B and 3C), coinciding with the limited induction of islet cell death and reduced frequency of diabetes induction in the RIP-gp model. Together, these data demonstrate that HIF-1α-Tg BMDC vaccination results in suboptimal activation of CD8^+^ T cells *in vivo*.

To further demonstrate that HIF-1α-Tg BMDCs had a negative impact on their ability to activate CD8^+^ T cells, we measured the lysis of peptide-pulsed targets *in vivo*. WT mice were immunized with WT or HIF-1α-Tg BMDCs that had been pulsed with LCMV gp peptides and stimulated with CpG to induce an antigen-specific immune response. Eight days later, a 1:1 mixture of labeled, gp33 peptide-pulsed and adenoviral control peptide-pulsed targets were infused via tail vein injection into the same WT mice. The spleens of these mice were harvested several hours later and analyzed by flow cytometry to assess specific killing of labeled target cells (Fig 3D). We found that HIF-1α-Tg BMDCs induced a suboptimal cytotoxic response compared to WT BMDCs (Fig 3E), and these data are consistent with the limited induction of diabetes observed earlier (Fig 2B and 2C).

To further confirm our findings that HIF-1α limits DC function, we tested HIF-1α KO BMDCs in the RIP-gp model. HIF-1α KO BMDCs were evaluated for their capacity to activate antigen-specific, cytotoxic T cells *in vivo* (Fig 3D). In contrast to our findings with HIF-1α-Tg BMDCs, HIF-1α KO BMDCs were found to promote an enhanced cytotoxic response compared to WT (Fig 3F), although diabetes incidence was comparable to WT (S3 Fig). Collectively, these observations demonstrate that constitutive expression of HIF-1α impairs the ability of BMDCs to promote the expansion of gp-specific CTLs, which show reduced granzyme B expression and diminished cytotoxic function, while deletion of HIF-1α in BMDCs can augment their immunogenicity.

### HIF-1α expression promotes the production of immunosuppressive modulators including arginase, nitric oxide and VEGF

**Fig 4:**
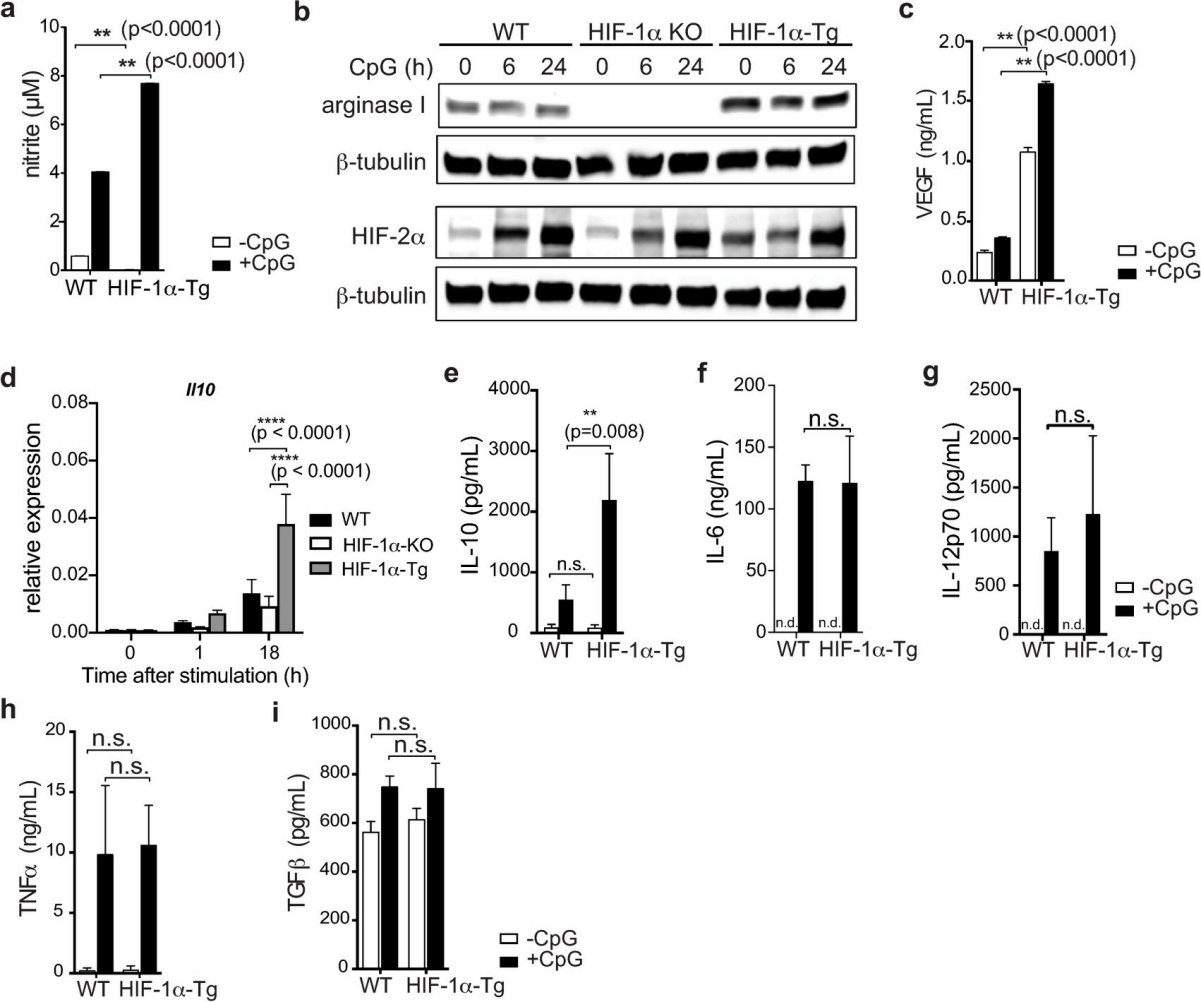
HIF-1α expression in BMDC cultures promotes the production of immunosuppressive cytokines. (**a**) Nitric oxide production by WT and HIF-1α-Tg BMDCs was indirectly quantified by measuring nitrite levels (Griess reaction) by ELISA. (**b**) Arginase I and HIF-2α protein levels in total WT and HIF-1α-Tg BMDC cell lysates. Cell lysates from the same samples were split across different gels and probed separately for arginase I and HIF-2α. β-tubulin was used as a protein loading control. (**c**) VEGF production by BMDCs was analyzed by ELISA. (**d**) *Il10* expression in BMDCs was analyzed by qPCR before and after CpG-stimulation. (**e**) IL-10, (**f**) IL-6, (**g**) IL-12p70, (**h**) TNFα, and (**i**) TGFβ production by BMDCs was analyzed by ELISA. All data are representative of two to three independent experiments. Error bars represent SD of the mean. Statistical testing was performed using two-way ANOVA and Tukey’s test for multiple comparison; n.s.: not significant, n.d.: not detected (below limit of detection).

We next asked whether HIF-1α could be mediating the production of immunosuppressive factors or limiting pro-inflammatory cytokine production that could potentially explain the reduced diabetes incidence observed with HIF-1α-Tg BMDC vaccination in the RIP-gp model. Since we detected increased expression of iNOS in HIF-1α-Tg BMDC cultures (Fig 1E), we analyzed additional targets of HIF-1α from the supernatants of unstimulated and CpG stimulated BMDC cultures. We observed elevated production of nitric oxide (measured as nitrite) and arginase I by HIF-1α-Tg BMDCs following stimulation compared to WT BMDCs (Fig 4A and 4B). Interestingly, although arginase I has been reported to be mainly a target of HIF-2α [30] in primary macrophages, we did not observe significantly altered levels of HIF-2α in HIF-1α-Tg BMDCs compared to WT (Fig 4B). Consistent with these data, arginase I was undetectable in HIF-1α KO BMDCs at both the transcript (S2 Fig) and protein level (Fig 4B), either before or after CpG stimulation, despite similar HIF-2α levels in HIF-1α KO compared to WT, suggesting that HIF-1α may play a predominant role in regulating arginase I expression in BMDCs. We also observed that levels of vascular endothelial growth factor (VEGF)–which is known to have suppressive properties on some immune cell subsets [31–33]–was found to be significantly upregulated in the supernatants of cultures of HIF-1α-Tg BMDCs following CpG stimulation compared to WT BMDCs (Fig 4C). *Il10*, which has been reported to be a direct transcriptional target of HIF-1α [34], was found to be significantly increased by qPCR (Fig 4D), and secreted IL-10 was also found to be significantly upregulated by stimulated HIF-1α-Tg BMDCs compared to WT (Fig 4E). Conversely, IL-6, IL-12p70, TNFα and TGFβ were not significantly altered with enforced HIF-1α expression in BMDCs at the protein (Fig 4F–4I) or transcript level (S4 Fig). Overall, these data indicate that HIF-1α drives multiple inhibitory mechanisms that contribute to limiting the induction of immunity by DCs.

## Discussion

Our study aimed to examine the effects of HIF-1α in dendritic cells by constitutive expression of a form of HIF-1α that cannot be recognized and targeted for degradation by pVHL. Previous studies examining the effects of HIF-1α on DC function have used conditional knockout approaches [35], culture in hypoxia [17], or small molecules such as cobalt chloride to mimic hypoxic conditions. These and other studies have yielded at times opposing data on the effects of HIF-1α on DCs. Some of these differences could be attributed to the experimental model tested, while HIF-1α-independent effects of hypoxia [36, 37], for example, could also explain some of the conflicting data in the literature.

We generated and evaluated a novel system where HIF-1α was conditionally and constitutively expressed in BMDCs. Our data demonstrate that HIF-1α expression in BMDCs limited their ability to activate a cytotoxic T cell response *in vivo*. We found that HIF-1α expression did not alter the surface levels of co-stimulatory markers or significantly alter the production of pro-inflammatory cytokines by BMDCs. While previous studies have shown that hypoxia can induce upregulation of CD80, CD86 and MHCII [13, 35] in BMDCs, we did not observe these effects with constitutive expression of HIF-1α. It is likely that hypoxia-driven, but HIF-1α-independent pathways, such as NF-κB [37, 38], were responsible for the phenotypes observed in these studies.

Our findings suggest that constitutive expression of HIF-1α in BMDCs promotes the production of several immune inhibitory mediators. HIF-1α has been shown to directly increase the transcription of the genes for many of these molecules, including *Il10* [34], *VEGFA* [39], *NOS2* [40] and *ARG1* [41]. Consistent with these data, we observed increased levels of IL-10, VEGF, iNOS, and arginase in HIF-1α-Tg BMDC cultures. Interestingly, *Arg1* has been identified to be mainly regulated by HIF-2α [30], while our data suggest that HIF-1α, and not HIF-2α, is the predominant driver of *Arg1* expression in BMDCs. The reason(s) for the pre-dominant role of HIF-1α in regulating *Arg1* expression in BMDCs is unclear, although the same study showed that HIF-1α can also be involved in regulating *Arg1* expression as conditional deletion of HIF-1α in macrophages resulted in reduced expression of *Arg1*. While HIF-1α and HIF-2α bind to the same hypoxia responsive element (HRE) [42], and can have overlapping targets, the divergence in their regulatory programs has been associated with their interaction with other transcription factors and co-regulators [43]. The precise co-factors that cooperate with HIF-1α to drive *Arg1* expression in BMDCs will require further investigation to clarify the context-specific roles of HIF-1α and HIF-2α in regulating *Arg1* expression. Overall, our data supports the notion that HIF-1α functions as a crucial transcription factor that drives the production of immunosuppressive molecules. Although we did not identify the specific factor(s) responsible for reduced DC function in our model, it is likely that a combination of factors driven by HIF-1α collectively contributes to inhibiting DC activation of effector T cells.

Consistent with previous reports [14, 44], we also found that HIF-1α was upregulated following TLR stimulation. Several different, but potentially overlapping mechanisms have been described to link increased HIF-1α levels following TLR activation, including the activation of NF-κB [44] and/or ERK signaling [45] pathways, the generation of reactive oxygen species [46], succinate [14], or the sequestration of iron [47] (a co-factor for the PHD family). A later study suggested that in contrast to studies using mouse cells, a p38-MAPK-dependent pathway was important for HIF-1α stabilization in human monocyte-derived DCs following stimulation with LPS [48]. While the dominant mechanism for HIF-1α upregulation downstream of TLR signaling remains to be fully delineated, these data show that increased HIF-1α is a common effect of TLR stimulation of DCs.

Although HIF-1α has been associated with both pro- and anti-inflammatory functions of DCs, we show that conditional deletion of HIF-1α in BMDCs was able to significantly enhance the generation of antigen-specific, cytotoxic T cell responses *in vivo*. These findings further support work from other groups showing that HIF-1α in DCs limited the expansion of CD8^+^ T cells and the downstream induction of KLRG1^+^ CD8^+^ short-lived effector T cells (SLECs) in a *Leishmania* infection model [20].

It is important to note that the kinetics of HIF-1α also appears to be cell type-specific. HIF-1α has been shown to be rapidly upregulated upon exposure to hypoxia, but subsequently degraded 4-8h later despite the maintenance of hypoxic conditions [49, 50]. In contrast, HIF-1α expression has been observed to remain upregulated following 6 days of culture of monocyte-derived DCs in hypoxic conditions [17]. These and subsequent studies will benefit from the development of inducible models capable of both temporal and conditional expression of HIF-1α *in vivo*, and would help to refine our understanding of the functions of HIF-1α in immune cells.

The role of myeloid cells in promoting tumor growth and dampening anti-tumor responses has been well established [51–53]. Our data are consistent with previous reports suggesting that HIF-1α limits dendritic cell function [19, 21]. Taken together, these results suggest that constitutive HIF-1α activation in dendritic cells–for example, following migration within the hypoxic tumor microenvironment–might impair anti-tumor responses by limiting effector T cell cytotoxicity and function. Thus, treatments aiming to modulate HIF-1α activity will ideally consider the immune cell types that would be affected, and will enhance or inhibit HIF-1α function based on the desired therapeutic outcome.

## Materials and methods

### Generation of HIF-1α-Tg and HIF-1α KO mice

LSL-HIF-1a-dPA mice were obtained from The Jackson Laboratory (JAX stock 009673) and bred to *Vav-Cre* mice to generate HIF-1α-Tg mice. *Hif1a*^*fl/fl*^ mice (JAX stock 007561) were bred to *Cd11c-Cre*-*Gfp* mice (JAX stock 007567) to generate HIF-1α KO mice (*Hif-1a*^*fl/fl*^ *Cd11c-Cre-Gfp*). P14 and RIP-gp mice were previously described [27, 54]. All animal experiments were performed in the Ontario Cancer Institute (OCI) animal facility and with the approval of the OCI Animal Ethics Committee, under Animal User Protocol (AUP) 929.

### Diabetes experiments in the RIP-gp model

Bone-marrow derived dendritic cells were cultured from bone marrow harvested from the femurs and tibiae of mice. Additional details can be found in a previously published study from our laboratory [28]. Briefly, BM cells were seeded at 2 x 10^6^ cells per plate in 10cm plates and cultured for 8 days in the presence of GM-CSF. Non-adherent cells were harvested and either stimulated with 10μM ODN1826 CpG (Integrated DNA Technologies), or left unstimulated for 16-20h. Cells were then pulsed with a mixture of gp33-41 (KAVYNFATM), gp276-286 (SGVENPGGYCL) and gp61-80 (GLNGPDIYKGVYQFKSVEFD) peptides at a final concentration of 1μM for 3h, washed three times, and infused intravenously via tail-vein into RIP-gp mice. Blood glucose was monitored using Accu-Chek Performa (Roche) blood glucose meters and Accu-Chek Inform II (Roche) blood glucose test strips.

### *In vivo* cytotoxic T lymphocyte assay

B6 mice were vaccinated with 2 x 10^6^ peptide-pulsed BMDCs as described above. Eight days after vaccination, a 1:1 ratio of splenocytes pulsed with gp33-41 or a control AV peptide (SGPSNTPPEI), labeled with e450 proliferation dye (eBioscience) at 40μM or 5μM, respectively, were injected via tail vein. Approximately 1 x 10^7^ splenocytes were injected per mouse. Four hours after injection, mice were sacrificed and the spleen harvested and homogenized for flow cytometry analysis. Specific killing was calculated using the following formula: (percentage AV–percentage gp33-41) / (percentage AV) x 100%.

### Flow cytometry

Flow cytometry data was acquired using the BD FACS Canto II (Becton-Dickinson) or BD LSR Fortessa (Becton-Dickinson). Data analysis was performed using Flowjo (Treestar Software). Antibodies were purchased from BD Biosciences, Affymetrix/eBioscience and Biolegend. Antibodies for CCR7 (4B12), CD11b (M1/70), CD11c (N418), CD44 (IM7), CD115 (AFS98), F4/80 (BM8), Gr-1 (RB6-8C5), Ly-6C (HK1.4), Ly-6G (1A8-Ly6G), MHC I (34-1-2S), OX40L (RM134L), PD-1 (J43), PD-L1 (MIH5), PD-L2 (TY25) were purchased from eBioscience. CD25 (PC61), CD40 (3/23), CD80 (16-10A1), CD103 (M290), and annexin V antibodies were from BD Biosciences. 7AAD was also from BD Biosciences. CD86 (GL-1) and MHC II (M5/114.15.2) antibodies were from Biolegend. gp34-tetramer was prepared by sequential addition of streptavidin APC or streptavidin-R-PE (Invitrogen) to biotinylated gp34 monomers (H-2K^b^; AVYNFATC) generously provided by the NIH Tetramer Core Facility.

### ELISA for cytokines

BMDC supernatants were tested for VEGF, IL-6, IL-10, IL-12p70, IL-23, and TNFα by ELISA, with appropriate dilutions. The VEGF ELISA kit was purchased from Peprotech, and the other ELISA kits were purchased from eBioscience (Ready-SET-Go!, Thermo Fisher Scientific). Supernatants were diluted as necessary according to the kit manufacturer’s instructions.

### Quantitative PCR

Total RNA was extracted from BMDCs using a RNeasy Plus Mini Kit (Qiagen) or TRIzol reagent (Thermo Fisher Scientific). cDNA was synthesized using a high capacity cDNA reverse transcription kit (Applied Biosystems, Thermo Fisher Scientific) according to the manufacturer’s instructions. Quantitative, real-time PCR was performed with KAPA SYBR (KAPA Biosystems) and an ABI 7900HT sequence detection system (Applied Biosystems). Actin was used as a housekeeping gene control to normalize target gene expression. Primer sequences can be found in S1 Table.

### Western blot

Protein samples were separated on 4–12% Bis-Tris acrylamide gels (Life Technologies) and transferred to PVDF (Millipore). HIF-1α antibody was purchased from Cayman Chemical (10006421) and Novus Bio (NB100-479). Arginase I (H-52) and NOS2 (M19) antibodies were purchased from Santa Cruz Biotechnology. Rabbit anti-β-actin (A2066) was purchased from Sigma. β-tubulin (AA2) was purchased from Millipore. Secondary antibodies were goat anti-rabbit HRP (Thermo), goat anti-mouse HRP (Thermo), donkey anti-mouse IRDye800CW, donkey anti-goat IRDye680RD and donkey anti-rabbit IRDye680RD (Licor). Blots were developed with Supersignal Femto (Thermo) or Amersham ECL (GE Healthcare Life Sciences) or visualized on an Odyssey CLx imager (Licor).

### Statistical methods

Statistical testing was performed in Graphpad Prism 7.0e. The threshold for statistical significance was set at alpha = 0.05. Corrections were applied to multiple comparisons testing as indicated in the figure legends.

## Supporting information

S1 Fig Cell viability and antigen uptake and processing are similar between WT and HIF-1α-Tg BMDCs. (**a**) WT or HIF-1α-Tg BMDCs were stained with 7AAD and anti-annexin V and analyzed by flow cytometry to assess viability at d8 of BMDC culture. (**b**) BMDCs were incubated in the presence of OVA-Alexa488 and with or without CpG for 16h. Cells were stained and analyzed by flow cytometry for the uptake of OVA-Alexa488 (indicated as percent positive). (**c**) Unstimulated or (**d**) CpG-stimulated WT or HIF-1α-Tg BMDCs were cultured with DQ-OVA at 0°C (white bars) or 37°C (black bars) for 1h, and DQ-OVA uptake and processing quantified by flow cytometry. Processing and cleavage of DQ-OVA antigen processing is indicated by an increase in mean fluorescence intensity. Data are representative of two independent experiments. n.s.: not significant. Statistical testing was performed with two-way ANOVA and Tukey’s test.
(TIF)

S2 Fig Expression of selected HIF-1α target genes in WT, HIF-1α KO and HIF-1α-Tg BMDCs. (**a**) WT, HIF-1**α** KO and HIF-1**α**-Tg BMDCs were stimulated for various durations with CpG. Cells were harvested following stimulation and analyzed by qPCR for the expression of the indicated genes. Data are combined from a total of five biological replicates. Error bars represent standard error of the mean. For figure clarity, all pairwise comparisons were performed, but only statistically significant results are indicated. n.s: not significant. Statistical testing was performed with two-way ANOVA and Tukey’s test.
(TIF)

S3 Fig HIF-1α KO BMDCs have similar diabetes induction and expansion of gp34-tetramer specific CD8^+^ T cells in RIP-gp mice. (a) Cumulative diabetes incidence in RIP-gp mice receiving B6 or HIF-1α KO, CpG-stimulated, gp peptide-pulsed BMDCs. Data are representative of 18–23 mice per group. (b) RIP-gp mice were vaccinated with gp-peptide pulsed, unstimulated or CpG-stimulated, WT or HIF-1α KO BMDCs. Six days later, blood was collected for tetramer staining of gp34-tetramer-specific CD8^+^ T cells. n.s.: not significant. Error bars represent S.D. For (a), a log-rank (Mantel-Cox) test was used to determine significance, and for (b), a one-way ANOVA was performed with Tukey’s test for multiple comparisons.
(TIF)

S4 Fig Expression of pro-inflammatory cytokine genes in WT, HIF-1α KO and HIF-1α-Tg BMDCs. (**a**) WT, HIF-1**α** KO and HIF-1**α**-Tg BMDCs were stimulated for 0, 1 or 18h with CpG. Cells were harvested following stimulation and analyzed by qPCR for the expression of several commonly expressed, pro-inflammatory cytokine genes. Data are combined from a total of five biological replicates. Error bars represent standard error of the mean. For figure clarity, all pairwise comparisons were performed, but most comparison results with p > 0.05 are not indicated. n.s: not significant. Statistical testing was performed with two-way ANOVA and Tukey’s test.
(TIF)

S1 Table Sequences of primers used for gene expression profiling by quantitative PCR. (DOCX)

S1 Raw images (PDF)

## References

[1] Semenza GL. Hypoxia-Inducible Factors in Physiology and Medicine. Cell. Elsevier Inc; 2012;148: 399–408. 10.1016/j.cell.2012.01.021 22304911 PMC3437543

[2] Wang GL, Jiang BH, Rue EA, Semenza GL. Hypoxia-inducible factor 1 is a basic-helix-loop-helix-PAS heterodimer regulated by cellular O2 tension. Proc Natl Acad Sci USA. National Academy of Sciences; 1995;92: 5510–5514. 10.1073/pnas.92.12.5510 7539918 PMC41725

[3] Epstein AC, Gleadle JM, McNeill LA, Hewitson KS, O'Rourke J, Mole DR, et al C. elegans EGL-9 and mammalian homologs define a family of dioxygenases that regulate HIF by prolyl hydroxylation. Cell. 2001;107: 43–54. 10.1016/s0092-8674(01)00507-4 11595184

[4] Bruick RK, McKnight SL. A Conserved Family of Prolyl-4-Hydroxylases That Modify HIF. Science. American Association for the Advancement of Science; 2001;294: 1337–1340. 10.1126/science.1066373 11598268

[5] Ivan M, Haberberger T, Gervasi DC, Michelson KS, Günzler V, Kondo K, et al Biochemical purification and pharmacological inhibition of a mammalian prolyl hydroxylase acting on hypoxia-inducible factor. Proc Natl Acad Sci USA. National Acad Sciences; 2002;99: 13459–13464. 10.1073/pnas.192342099 12351678 PMC129695

[6] Berra E, Benizri E, Ginouvès A, Volmat V, Roux D, Pouysségur J. HIF prolyl‐hydroxylase 2 is the key oxygen sensor setting low steady‐state levels of HIF‐1α in normoxia. EMBO J. EMBO Press; 2003;22: 4082–4090. 10.1093/emboj/cdg392 12912907 PMC175782

[7] Maxwell PH, Wiesener MS, Chang GW, Clifford SC, Vaux EC, Cockman ME, et al The tumour suppressor protein VHL targets hypoxia-inducible factors for oxygen-dependent proteolysis. Nature. 1999;399: 271–275. 10.1038/20459 10353251

[8] Cramer T, Yamanishi Y, Clausen BE, Förster I, Pawlinski R, Mackman N, et al HIF-1alpha is essential for myeloid cell-mediated inflammation. Cell. 2003;112: 645–657. 10.1016/s0092-8674(03)00154-5 12628185 PMC4480774

[9] Bhandari T, Olson J, Johnson RS, Nizet V. HIF-1α influences myeloid cell antigen presentation and response to subcutaneous OVA vaccination. J Mol Med. Springer Berlin Heidelberg; 2013;91: 1199–1205. 10.1007/s00109-013-1052-y 23686259 PMC3783576

[10] Berger EA, McClellan SA, Vistisen KS, Hazlett LD. HIF-1α is essential for effective PMN bacterial killing, antimicrobial peptide production and apoptosis in Pseudomonas aeruginosa keratitis. PLoS Pathog. Public Library of Science; 2013;9: e1003457 10.1371/journal.ppat.1003457 23874197 PMC3715414

[11] Peyssonnaux C, Datta V, Cramer T, Doedens A, Theodorakis EA, Gallo RL, et al HIF-1alpha expression regulates the bactericidal capacity of phagocytes. J Clin Invest. American Society for Clinical Investigation; 2005;115: 1806–1815. 10.1172/JCI23865 16007254 PMC1159132

[12] Toussaint M, Fievez L, Drion P-V, Cataldo D, Bureau F, Lekeux P, et al Myeloid hypoxia-inducible factor 1|[alpha]| prevents airway allergy in mice through macrophage-mediated immunoregulation. Mucosal Immunol. Nature Publishing Group; 2013;6: 485–497. 10.1038/mi.2012.88 22968421

[13] Jantsch J, Chakravortty D, Turza N, Prechtel AT, Buchholz B, Gerlach RG, et al Hypoxia and hypoxia-inducible factor-1 alpha modulate lipopolysaccharide-induced dendritic cell activation and function. J Immunol. American Association of Immunologists; 2008;180: 4697–4705. 10.4049/jimmunol.180.7.4697 18354193

[14] Tannahill GM, Curtis AM, Adamik J, Palsson-McDermott EM, McGettrick AF, Goel G, et al Succinate is an inflammatory signal that induces IL-1β through HIF-1α. Nature. 2013;496: 238–242. 10.1038/nature11986 23535595 PMC4031686

[15] Cheng S-C, Quintin J, Cramer RA, Shepardson KM, Saeed S, Kumar V, et al mTOR- and HIF-1α-mediated aerobic glycolysis as metabolic basis for trained immunity. Science. 2014;345: 1250684–1250684. 10.1126/science.1250684 25258083 PMC4226238

[16] Guak H, Habyan Al S, Ma EH, Aldossary H, Al-Masri M, Won SY, et al Glycolytic metabolism is essential for CCR7 oligomerization and dendritic cell migration. Nat Comms. Nature Publishing Group; 2018;9: 2463–12. 10.1038/s41467-018-04804-6 29941886 PMC6018630

[17] Mancino A, Schioppa T, Larghi P, Pasqualini F, Nebuloni M, Chen I-H, et al Divergent effects of hypoxia on dendritic cell functions. Blood. 2008;112: 3723–3734. 10.1182/blood-2008-02-142091 18694997

[18] Flück K, Breves G, Fandrey J, Winning S. Hypoxia-inducible factor 1 in dendritic cells is crucial for the activation of protective regulatory T cells in murine colitis. Mucosal Immunol. 2015 10.1038/mi.2015.67 26220168

[19] Lawless SJ, Kedia-Mehta N, Walls JF, McGarrigle R, Convery O, Sinclair LV, et al Glucose represses dendritic cell-induced T cell responses. Nat Comms. Nature Publishing Group; 2017;8: 15620–14. 10.1038/ncomms15620 28555668 PMC5459989

[20] Hammami A, Charpentier T, Smans M, Staeger S. IRF-5-Mediated Inflammation Limits CD8(+) T Cell Expansion by Inducing HIF-1 alpha and Impairing Dendritic Cell Functions during Leishmania Infection. Denkers EY, editor. PLoS Pathog. Public Library of Science; 2015;11 10.1371/journal.ppat.1004938 26046638 PMC4457842

[21] Hammami A, Abidin BM, Heinonen KM, Stäger S. HIF-1α hampers dendritic cell function and Th1 generation during chronic visceral leishmaniasis. Sci Rep. 2018;8: 3500 10.1038/s41598-018-21891-z 29472618 PMC5823892

[22] Liu J, Zhang X, Chen K, Cheng Y, Liu S, Xia M, et al CCR7 Chemokine Receptor-Inducible lnc-Dpf3 Restrains Dendritic Cell Migration by Inhibiting HIF-1α-Mediated Glycolysis. Immunity. 2019;50: 600–615.e15. 10.1016/j.immuni.2019.01.021 30824325

[23] Kim WY, Safran M, Buckley MRM, Ebert BL, Glickman J, Bosenberg M, et al Failure to prolyl hydroxylate hypoxia-inducible factor alpha phenocopies VHL inactivation in vivo. EMBO J. 2006;25: 4650–4662. 10.1038/sj.emboj.7601300 16977322 PMC1589988

[24] Wood SM, Wiesener MS, Yeates KM, Okada N, Pugh CW, Maxwell PH, et al Selection and analysis of a mutant cell line defective in the hypoxia-inducible factor-1 alpha-subunit (HIF-1alpha). Characterization of hif-1alpha-dependent and -independent hypoxia-inducible gene expression. J Biol Chem. American Society for Biochemistry and Molecular Biology; 1998;273: 8360–8368. 10.1074/jbc.273.14.8360 9525945

[25] Melillo G, Taylor LS, Brooks A, Musso T, Cox GW, Varesio L. Functional requirement of the hypoxia-responsive element in the activation of the inducible nitric oxide synthase promoter by the iron chelator desferrioxamine. J Biol Chem. 1997;272: 12236–12243. 10.1074/jbc.272.18.12236 9115299

[26] Noman MZ, Desantis G, Janji B, Hasmim M, Karray S, Dessen P, et al PD-L1 is a novel direct target of HIF-1α, and its blockade under hypoxia enhanced MDSC-mediated T cell activation. J Exp Med. 2014;211: 781–790. 10.1084/jem.20131916 24778419 PMC4010891

[27] Ohashi PS, Oehen S, Buerki K, Pircher H, Ohashi CT, Odermatt B, et al Ablation of “tolerance” and induction of diabetes by virus infection in viral antigen transgenic mice. Cell. Elsevier; 1991;65: 305–317. 10.1016/0092-8674(91)90164-t 1901764

[28] Dissanayake D, Hall H, Berg-Brown N, Elford AR, Hamilton SR, Murakami K, et al Nuclear factor-κB1 controls the functional maturation of dendritic cells and prevents the activation of autoreactive T cells. Nat Med. 2011;17: 1663–1667. 10.1038/nm.2556 22081022

[29] Lin ACC, Dissanayake D, Dhanji S, Elford AR, Ohashi PS. Different toll-like receptor stimuli have a profound impact on cytokines required to break tolerance and induce autoimmunity. Boudinot P, editor. PLoS ONE. 2011;6: e23940 10.1371/journal.pone.0023940 21931625 PMC3171407

[30] Takeda N, O'Dea EL, Doedens A, Kim J-W, Weidemann A, Stockmann C, et al Differential activation and antagonistic function of HIF-{alpha} isoforms in macrophages are essential for NO homeostasis. Genes Dev. Cold Spring Harbor Lab; 2010;24: 491–501. 10.1101/gad.1881410 20194441 PMC2827844

[31] Gabrilovich DI, Chen HL, Girgis KR, Cunningham HT, Meny GM, Nadaf S, et al Production of vascular endothelial growth factor by human tumors inhibits the functional maturation of dendritic cells. Nat Med. 1996;2: 1096–1103. 10.1038/nm1096-1096 8837607

[32] Ziogas AC, Gavalas NG, Tsiatas M, Tsitsilonis O, Politi E, Terpos E, et al VEGF directly suppresses activation of T cells from ovarian cancer patients and healthy individuals via VEGF receptor Type 2. Int J Cancer. John Wiley & Sons, Ltd; 2012;130: 857–864. 10.1002/ijc.26094 21445972

[33] Kaur S, Chang T, Singh SP, Lim L, Mannan P, Garfield SH, et al CD47 signaling regulates the immunosuppressive activity of VEGF in T cells. The Journal of Immunology. American Association of Immunologists; 2014;193: 3914–3924. 10.4049/jimmunol.1303116 25200950 PMC4185246

[34] Meng X, Grötsch B, Luo Y, Knaup KX, Wiesener MS, Chen X-X, et al Hypoxia-inducible factor-1α is a critical transcription factor for IL-10-producing B cells in autoimmune disease. Nat Comms. Nature Publishing Group; 2018;9: 251–17. 10.1038/s41467-017-02683-x 29343683 PMC5772476

[35] Köhler T, Reizis B, Johnson RS, Weighardt H, Förster I. Influence of hypoxia-inducible factor 1α on dendritic cell differentiation and migration. Eur J Immunol. 2012;42: 1226–1236. 10.1002/eji.201142053 22539295 PMC6592818

[36] Romero-Ramirez L, Cao H, Nelson D, Hammond E, Lee A-H, Yoshida H, et al XBP1 is essential for survival under hypoxic conditions and is required for tumor growth. Cancer Res. American Association for Cancer Research; 2004;64: 5943–5947. 10.1158/0008-5472.CAN-04-1606 15342372

[37] Culver C, Sundqvist A, Mudie S, Melvin A, Xirodimas D, Rocha S. Mechanism of hypoxia-induced NF-kappaB. Mol Cell Biol. American Society for Microbiology Journals; 2010;30: 4901–4921. 10.1128/MCB.00409-10 20696840 PMC2950552

[38] Koong AC, Chen EY, Mivechi NF, Denko NC, Stambrook P, Giaccia AJ. Hypoxic activation of nuclear factor-kappa B is mediated by a Ras and Raf signaling pathway and does not involve MAP kinase (ERK1 or ERK2). Cancer Res. 1994;54: 5273–5279. 7923153

[39] Forsythe JA, Jiang BH, Iyer NV, Agani F, Leung SW, Koos RD, et al Activation of vascular endothelial growth factor gene transcription by hypoxia-inducible factor 1. Mol Cell Biol. American Society for Microbiology; 1996;16: 4604–4613. 10.1128/mcb.16.9.4604 8756616 PMC231459

[40] Jung F, Palmer LA, Zhou N, Johns RA. Hypoxic regulation of inducible nitric oxide synthase via hypoxia inducible factor-1 in cardiac myocytes. Circ Res. 2000;86: 319–325. 10.1161/01.res.86.3.319 10679484

[41] Louis CA, Mody V, Henry WL, Reichner JS, Albina JE. Regulation of arginase isoforms I and II by IL-4 in cultured murine peritoneal macrophages. Am J Physiol. 1999;276: R237–42. 10.1152/ajpregu.1999.276.1.R237 9887201

[42] Schödel J, Oikonomopoulos S, Ragoussis J, Pugh CW, Ratcliffe PJ, Mole DR. High-resolution genome-wide mapping of HIF-binding sites by ChIP-seq. Blood. 2011;117: e207–17. 10.1182/blood-2010-10-314427 21447827 PMC3374576

[43] Hu C-J, Sataur A, Wang L, Chen H, Simon MC. The N-terminal transactivation domain confers target gene specificity of hypoxia-inducible factors HIF-1alpha and HIF-2alpha. Tansey W, editor. Mol Biol Cell. 2007;18: 4528–4542. 10.1091/mbc.e06-05-0419 17804822 PMC2043574

[44] Rius J, Guma M, Schachtrup C, Akassoglou K, Zinkernagel AS, Nizet V, et al NF-κB links innate immunity to the hypoxic response through transcriptional regulation of HIF-1α. Nature. 2008;453: 807–811. 10.1038/nature06905 18432192 PMC2669289

[45] Frede S, Stockmann C, Freitag P, Fandrey J. Bacterial lipopolysaccharide induces HIF-1 activation in human monocytes via p44/42 MAPK and NF-kappaB. Biochem J. 2006;396: 517–527. 10.1042/BJ20051839 16533170 PMC1482811

[46] Nicholas SA, Bubnov VV, Yasinska IM, Sumbayev VV. Involvement of xanthine oxidase and hypoxia-inducible factor 1 in Toll-like receptor 7/8-mediated activation of caspase 1 and interleukin-1β. Cell Mol Life Sci. SP Birkhäuser Verlag Basel; 2011;68: 151–158. 10.1007/s00018-010-0450-3 20632067 PMC11115034

[47] Siegert I, Schödel J, Nairz M, Schatz V, Dettmer K, Dick C, et al Ferritin-Mediated Iron Sequestration Stabilizes Hypoxia-Inducible Factor-1α upon LPS Activation in the Presence of Ample Oxygen. Cell Reports. 2015;13: 2048–2055. 10.1016/j.celrep.2015.11.005 26628374

[48] Perrin-Cocon L, Aublin-Gex A, Diaz O, Ramière C, Peri F, André P, et al Toll-like Receptor 4-Induced Glycolytic Burst in Human Monocyte-Derived Dendritic Cells Results from p38-Dependent Stabilization of HIF-1α and Increased Hexokinase II Expression. The Journal of Immunology. American Association of Immunologists; 2018;201: 1510–1521. 10.4049/jimmunol.1701522 30037846

[49] Stroka DM, Burkhardt T, Desbaillets I, Wenger RH, Neil DA, Bauer C, et al HIF-1 is expressed in normoxic tissue and displays an organ-specific regulation under systemic hypoxia. FASEB J. 2001;15: 2445–2453. 10.1096/fj.01-0125com 11689469

[50] Brugarolas JB, Vazquez F, Reddy A, Sellers WR, Kaelin WG. TSC2 regulates VEGF through mTOR-dependent and -independent pathways. Cancer Cell. Elsevier; 2003;4: 147–158. 10.1016/s1535-6108(03)00187-9 12957289

[51] Ugel S, De Sanctis F, Mandruzzato S, Bronte V. Tumor-induced myeloid deviation: when myeloid-derived suppressor cells meet tumor-associated macrophages. J Clin Invest. American Society for Clinical Investigation; 2015;125: 3365–3376. 10.1172/JCI80006 26325033 PMC4588310

[52] Noy R, Pollard JW. Tumor-associated macrophages: from mechanisms to therapy. Immunity. 2014;41: 49–61. 10.1016/j.immuni.2014.06.010 25035953 PMC4137410

[53] Marvel D, Gabrilovich DI. Myeloid-derived suppressor cells in the tumor microenvironment: expect the unexpected. J Clin Invest. 2015;125: 3356–3364. 10.1172/JCI80005 26168215 PMC4588239

[54] Pircher H, Michalopoulos EE, Iwamoto A, Ohashi PS, Baenziger J, Hengartner H, et al Molecular analysis of the antigen receptor of virus-specific cytotoxic T cells and identification of a new V alpha family. Eur J Immunol. 1987;17: 1843–1846. 10.1002/eji.1830171226 2961577

