# Supplementary figures and images for: Hypoxia-inducible factor 1 alpha limits dendritic cell stimulation of CD8 T cell immunity
Source: PLoS One. 2020 Dec 31;15(12):e0244366. doi: 10.1371/journal.pone.0244366 (PMC7775062; doi:10.1371/journal.pone.0244366)

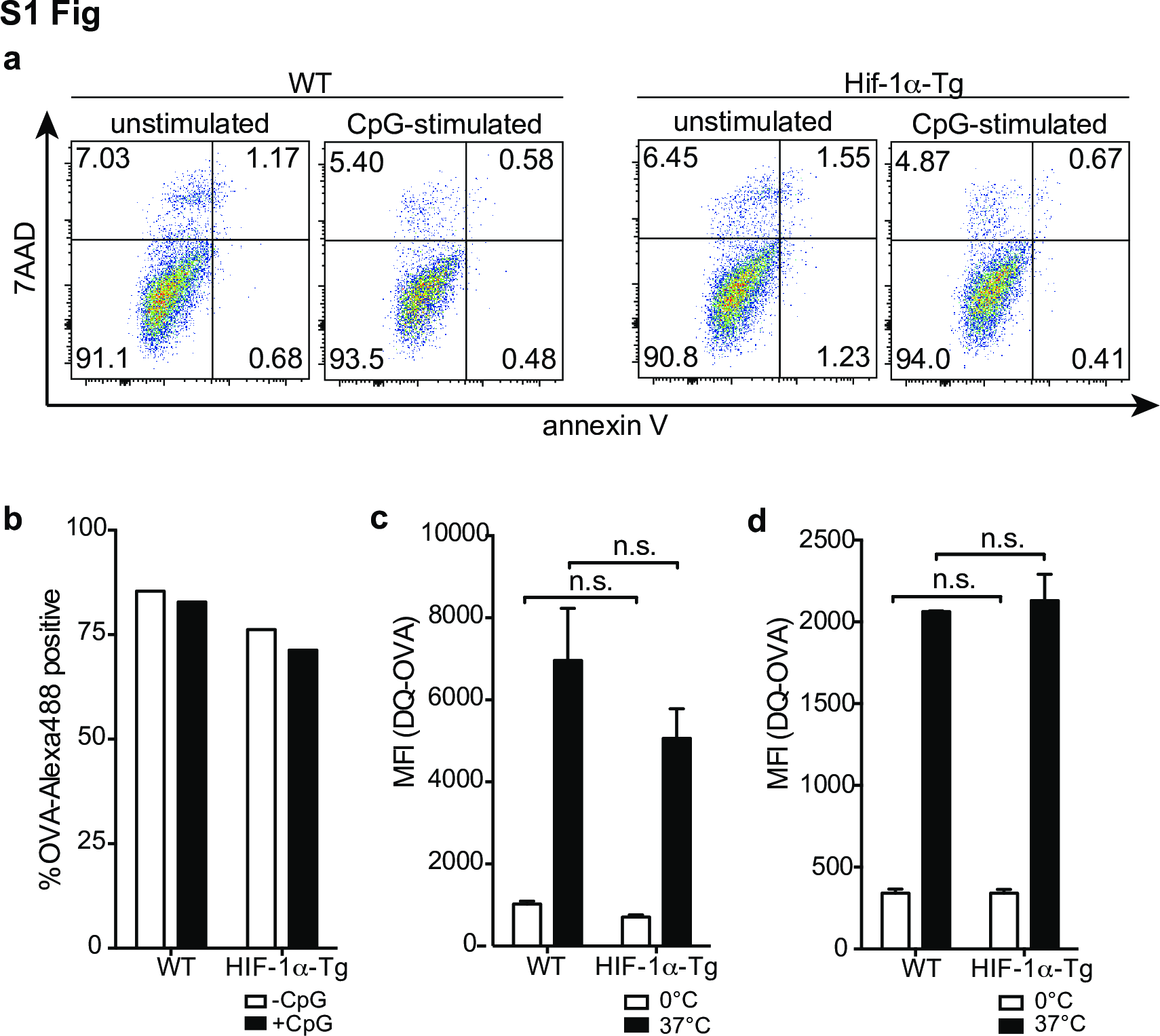

Supplement: S1 Fig — (a) WT or HIF-1α-Tg BMDCs were stained with 7AAD and anti-annexin V and analyzed by flow cytometry to assess viability at d8 of BMDC culture. (b) BMDCs were incubated in the presence of OVA-Alexa488 and with or without CpG for 16h. Cells were stained and analyzed by flow cytometry for the uptake of OVA-Alexa488 (indicated as percent positive). (c) Unstimulated or (d) CpG-stimulated WT or HIF-1α-Tg BMDCs were cultured with DQ-OVA at 0°C (white bars) or 37°C (black bars) for 1h, and DQ-OVA uptake and processing quantified by flow cytometry. Processing and cleavage of DQ-OVA antigen processing is indicated by an increase in mean fluorescence intensity. Data are representative of two independent experiments. n.s.: not significant. Statistical testing was performed with two-way ANOVA and Tukey’s test. (TIF) [file pone.0244366.s001.tif]

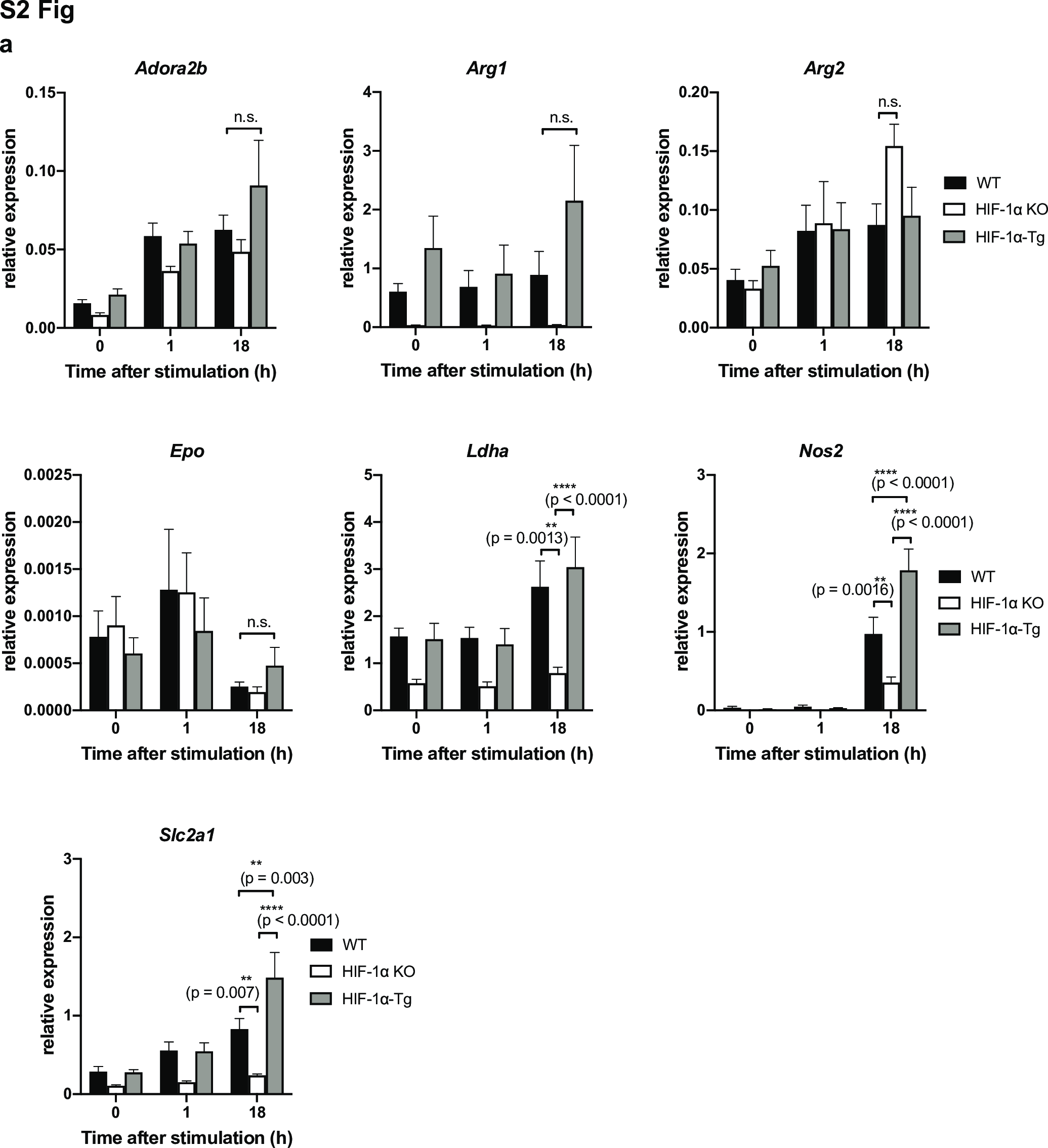

Supplement: S2 Fig — (a) WT, HIF-1α KO and HIF-1α-Tg BMDCs were stimulated for various durations with CpG. Cells were harvested following stimulation and analyzed by qPCR for the expression of the indicated genes. Data are combined from a total of five biological replicates. Error bars represent standard error of the mean. For figure clarity, all pairwise comparisons were performed, but only statistically significant results are indicated. n.s: not significant. Statistical testing was performed with two-way ANOVA and Tukey’s test. (TIF) [file pone.0244366.s002.tif]

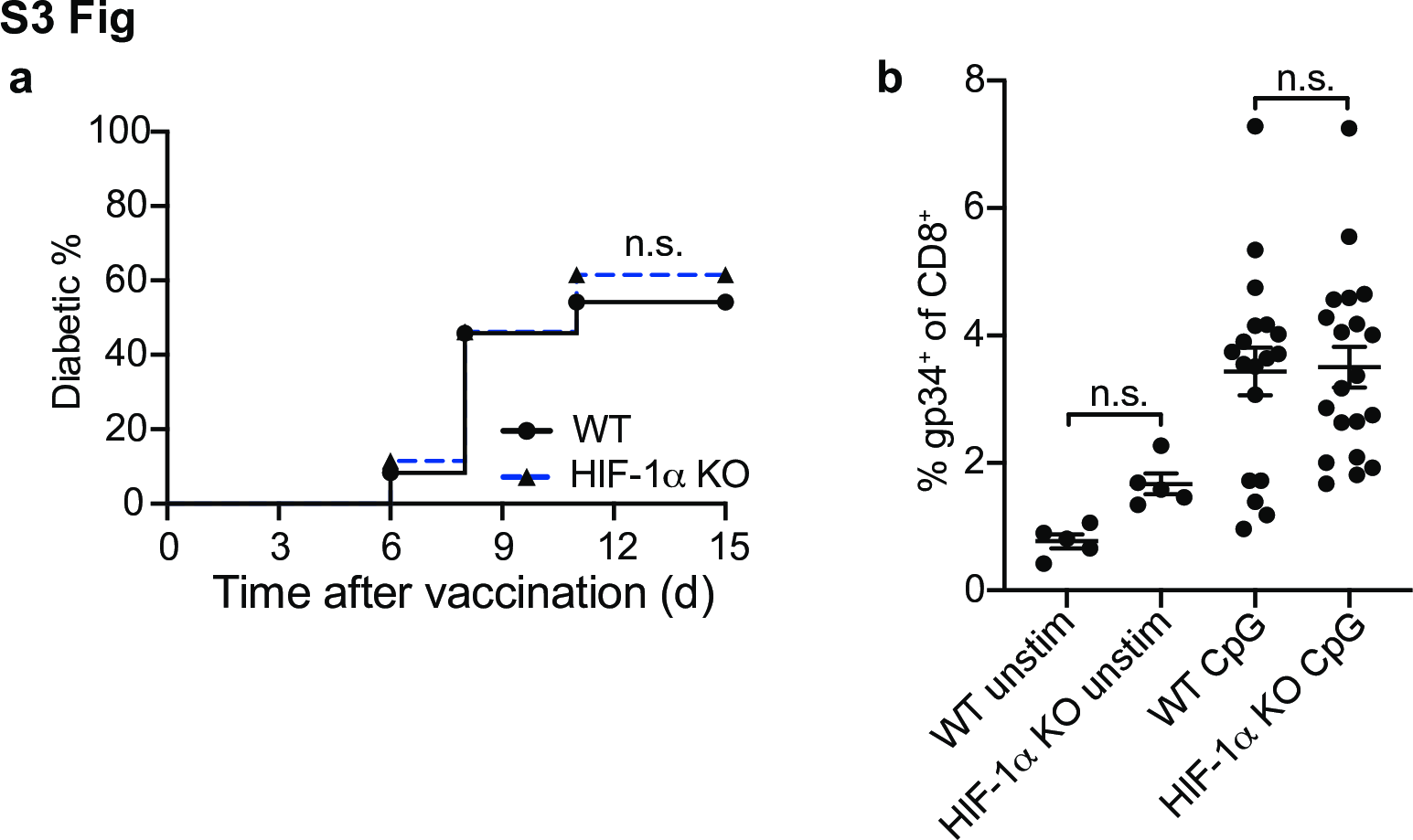

Supplement: S3 Fig — (a) Cumulative diabetes incidence in RIP-gp mice receiving B6 or HIF-1α KO, CpG-stimulated, gp peptide-pulsed BMDCs. Data are representative of 18–23 mice per group. (b) RIP-gp mice were vaccinated with gp-peptide pulsed, unstimulated or CpG-stimulated, WT or HIF-1α KO BMDCs. Six days later, blood was collected for tetramer staining of gp34-tetramer-specific CD8+ T cells. n.s.: not significant. Error bars represent S.D. For (a), a log-rank (Mantel-Cox) test was used to determine significance, and for (b), a one-way ANOVA was performed with Tukey’s test for multiple comparisons. (TIF) [file pone.0244366.s003.tif]

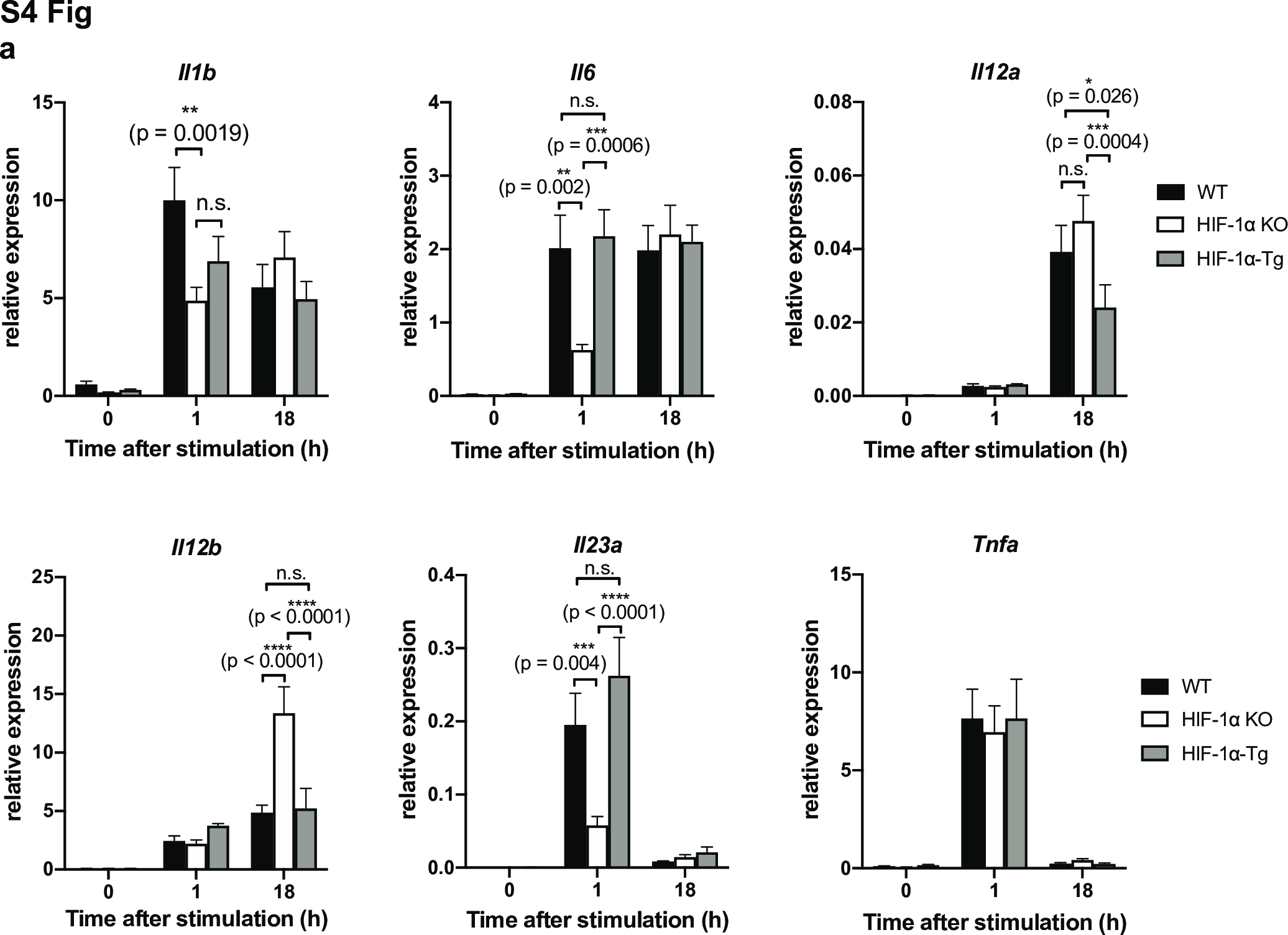

Supplement: S4 Fig — (a) WT, HIF-1α KO and HIF-1α-Tg BMDCs were stimulated for 0, 1 or 18h with CpG. Cells were harvested following stimulation and analyzed by qPCR for the expression of several commonly expressed, pro-inflammatory cytokine genes. Data are combined from a total of five biological replicates. Error bars represent standard error of the mean. For figure clarity, all pairwise comparisons were performed, but most comparison results with p > 0.05 are not indicated. n.s: not significant. Statistical testing was performed with two-way ANOVA and Tukey’s test. (TIF) [file pone.0244366.s004.tif]
