# Supplementary material for: Hypoxia-inducible factor 1 alpha limits dendritic cell stimulation of CD8 T cell immunity
Source: PLoS One. 2020 Dec 31;15(12):e0244366. doi: 10.1371/journal.pone.0244366 (PMC7775062; doi:10.1371/journal.pone.0244366)
Supplement: S1 Table — (DOCX) [file pone.0244366.s005.docx]

**S1 Table**

| **Gene** | **Forward primer (5’-3’)** | **Reverse primer (5’-3’)** | **Reference** |
| --- | --- | --- | --- |
| *Il1b* | AAGGAGAACCAAGCAACGACAAAA | TGGGGAACTCTGCAGACTCAAACT |  |
| *Il6* | TAG TCC TTC CTA CCC CAA TTT CC | TTG GTC CTT AGC CAC TCC TTC | Primerbank (13624311a1) |
| *Il10* | CTTACTGACTGGCATGAGGATCA | GCAGCTCTAGGAGCATGTGG | Primerbank (29157514c1) |
| *Il12a* | CAATCACGCTACCTCCTCTTTT | CAGCAGTGCAGGAATAATGTTTC | Primerbank (226874944c1) |
| *Il12b* | TGGTTTGCCATCGTTTTGCTG | ACAGGTGAGGTTCACTGTTTCT | Primerbank (6680397a1) |
| *Il23a* | CAGCAGCTCTCTCGGAATCTC | TGGATACGGGGCACATTATTTTT | primerbank (ID#: 133892789c1) |
| *Tnfa* | CATCTTCTCAAAATTCGAGTGACAA | TGGGAGTAGACAAGGTACAACCC |  |
| *Adora2b* | CTCACACAGAGCTCCATCTTTAG | GTCCCAGTGACCAAACCTTTA | Designed using IDT Primerquest tool |
| *Arg1* | CTCCAAGCCAAAGTCCTTAGAG | AGGAGCTGTCATTAGGGACATC | Zhou et al. PNAS 2011 Nov1;108(44):E998-1006) |
| *Arg2* | TCCTCCACGGGCAAATTCC | GCTGGACCATATTCCACTCCTA | Primerbank (6753110a1) |
| *Epo* | ACTCTCCTTGCTACTGATTCCT | ATCGTGACATTTTCTGCCTCC | Primerbank (21389309a1) |
| *Ldha* | TGTCTCCAGCAAAGACTACTGT | GACTGTACTTGACAATGTTGGGA | Guak et al. Nat Commun. 2018 Jun 25;9(1):2463 |
| *Nos2* | CCCTCCTGATCTTGTGTTGGA | CAACCCGAGCTCCTGGAAC | Zhou et al. PNAS 2011 Nov1;108(44):E998-1006) |
| *Slc2a1* | CTGGACCTCAAACTTCATTGTGGG | GGGTGTCTTGTCACTTTGGCTGG | Guak et al. Nat Commun. 2018 Jun 25;9(1):2463 |
| *Actb* | GGCTGTATTCCCCTCCATCG | CCAGTTGGTAACAATGCCATGT | Primerbank (6671509a1) |
