# Supplementary material for: Hypoxia-inducible factor 1 alpha limits dendritic cell stimulation of CD8 T cell immunity
Source: PLoS One. 2020 Dec 31;15(12):e0244366. doi: 10.1371/journal.pone.0244366 (PMC7775062; doi:10.1371/journal.pone.0244366)
Supplement: S1 Raw images — (PDF) [file pone.0244366.s006.pdf]

Fig 1d: HIF-1α

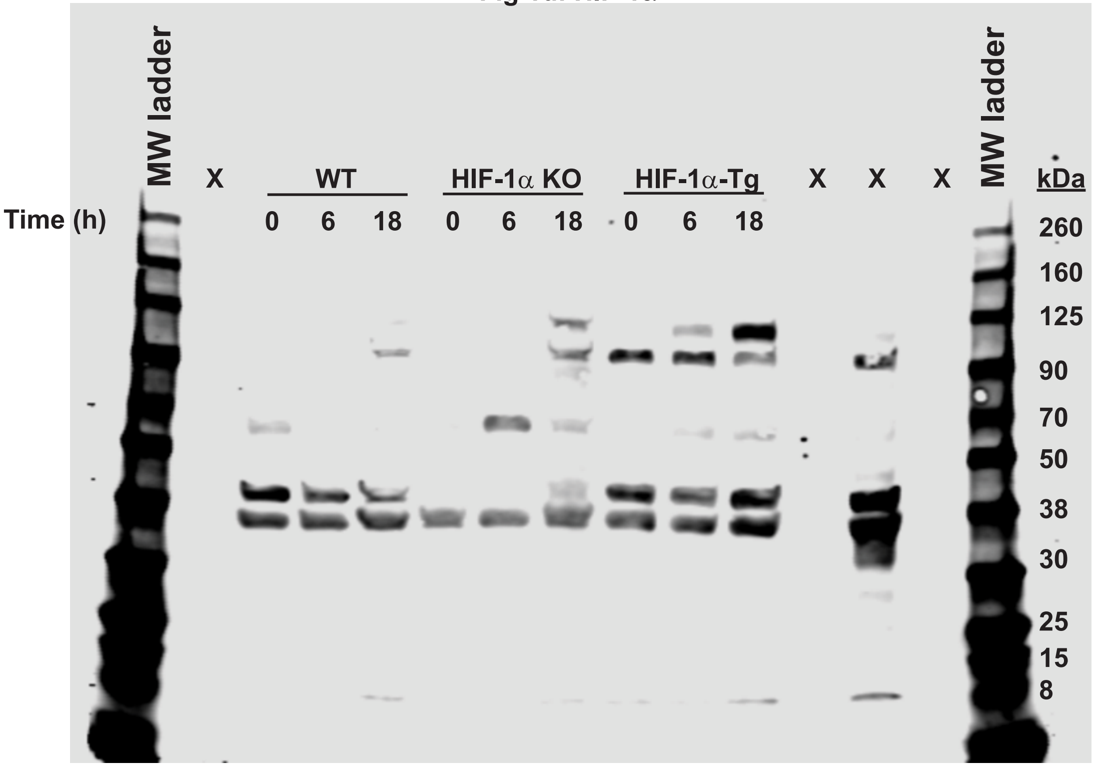

Fig 1d: β-tubulin

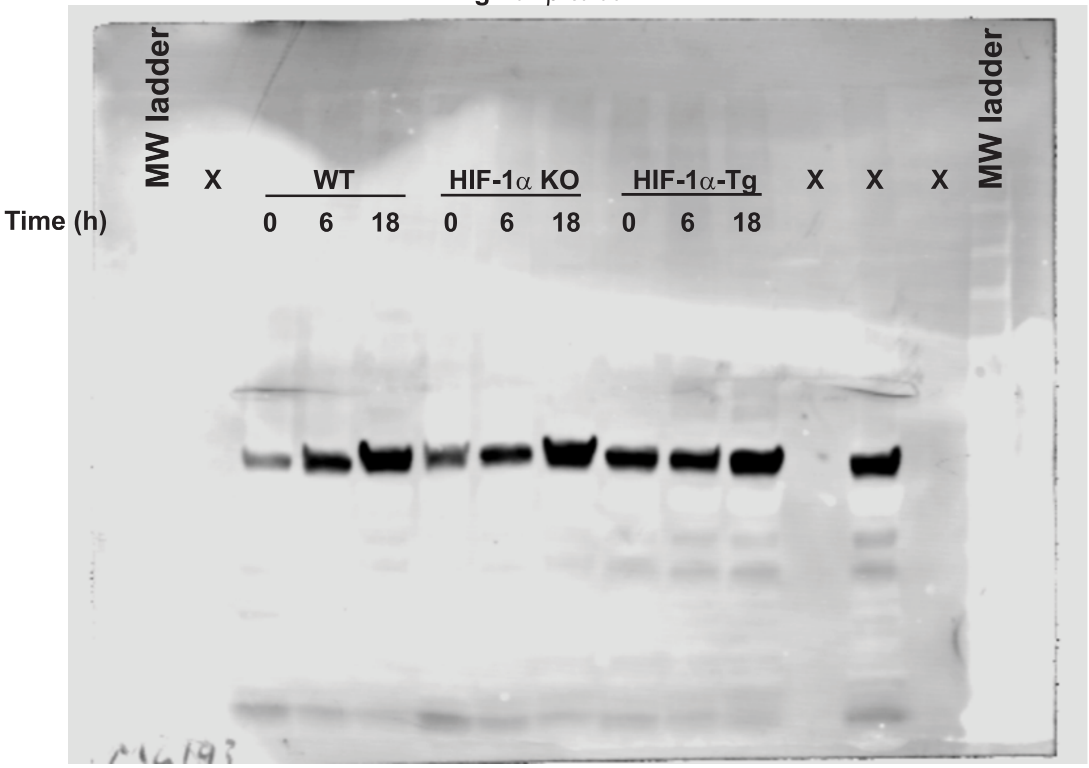

Both images for Fig 1d were captured using the Licor Odyssey CLx imaging system with Image Studio software.

Fig 1e: iNOS

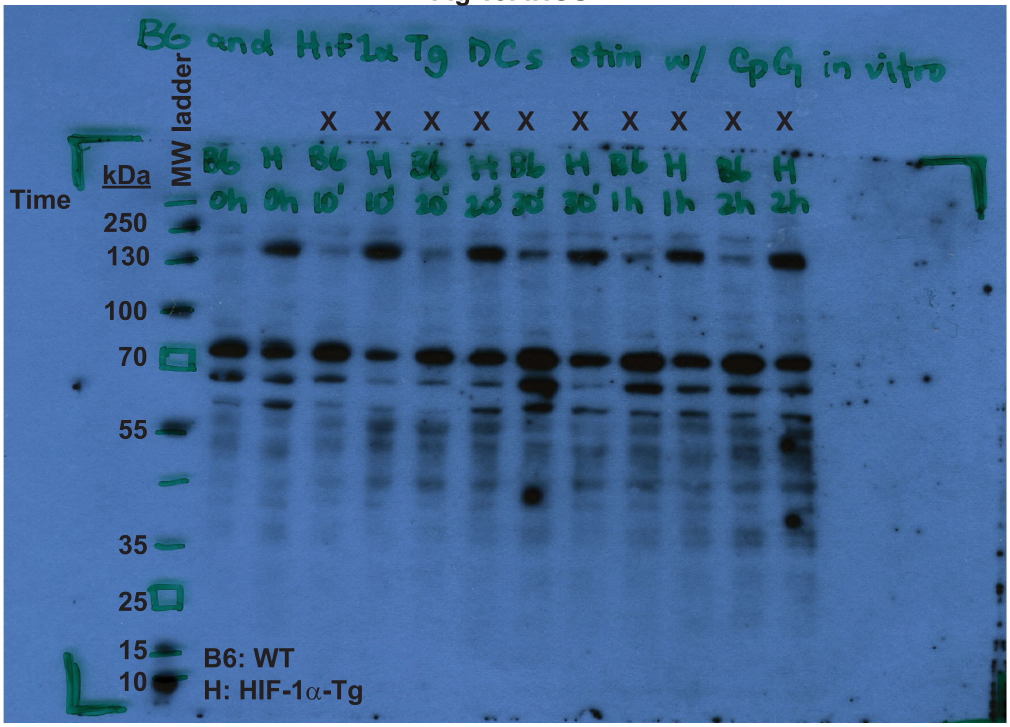

Fig 1e: β-actin

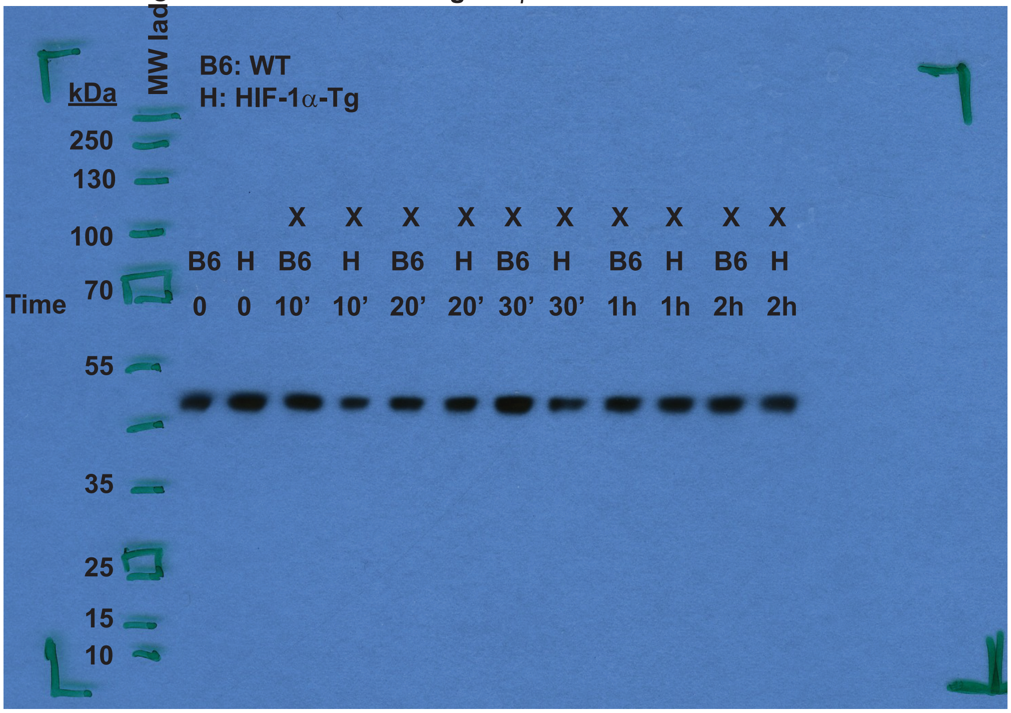

Images for Fig 1e were first captured on X-ray film after chemiluminescent development of the blot (Supersignal West Femto). Developed film was then scanned on a flatbed scanner to generate the digital images above.

Fig 4b (upper): arginase I

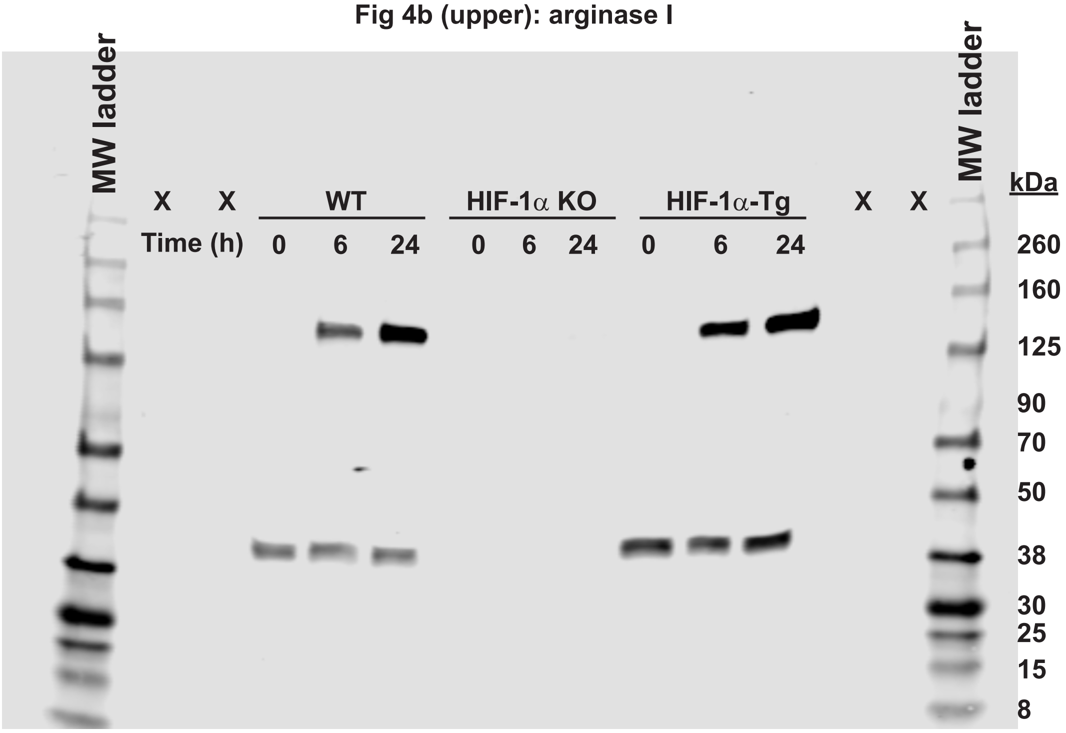

Fig 4b (upper): β-tubulin

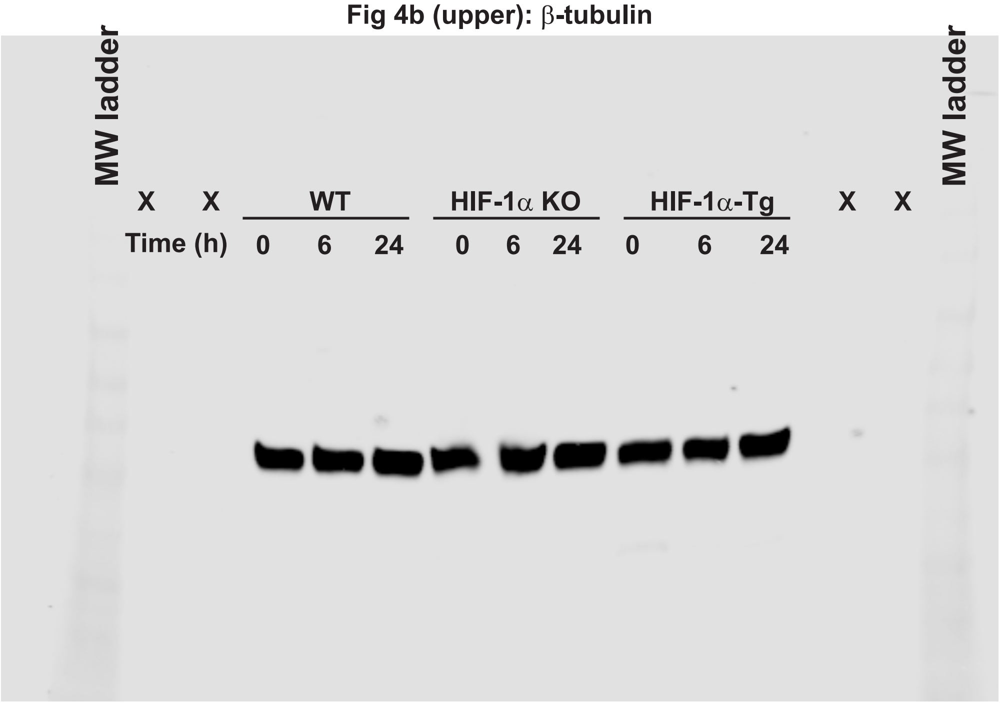

Both images shown above were captured using the Licor Odyssey CLx imaging system with Image Studio software.

Fig 4b (lower): HIF-2α

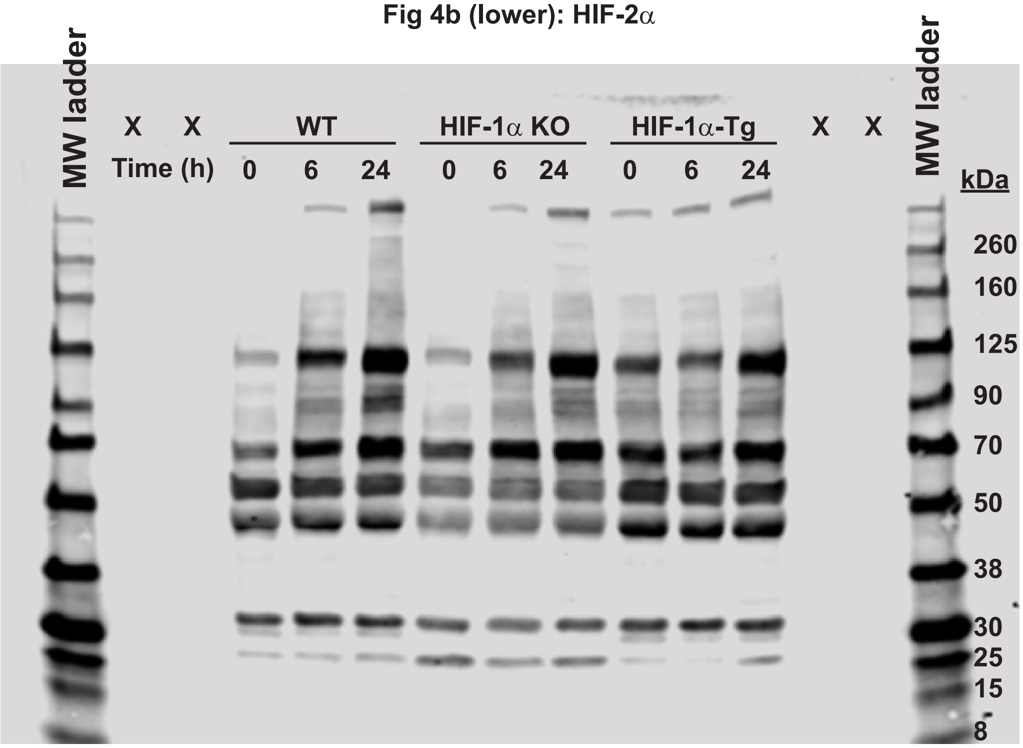

Fig 4b (lower): β-tubulin

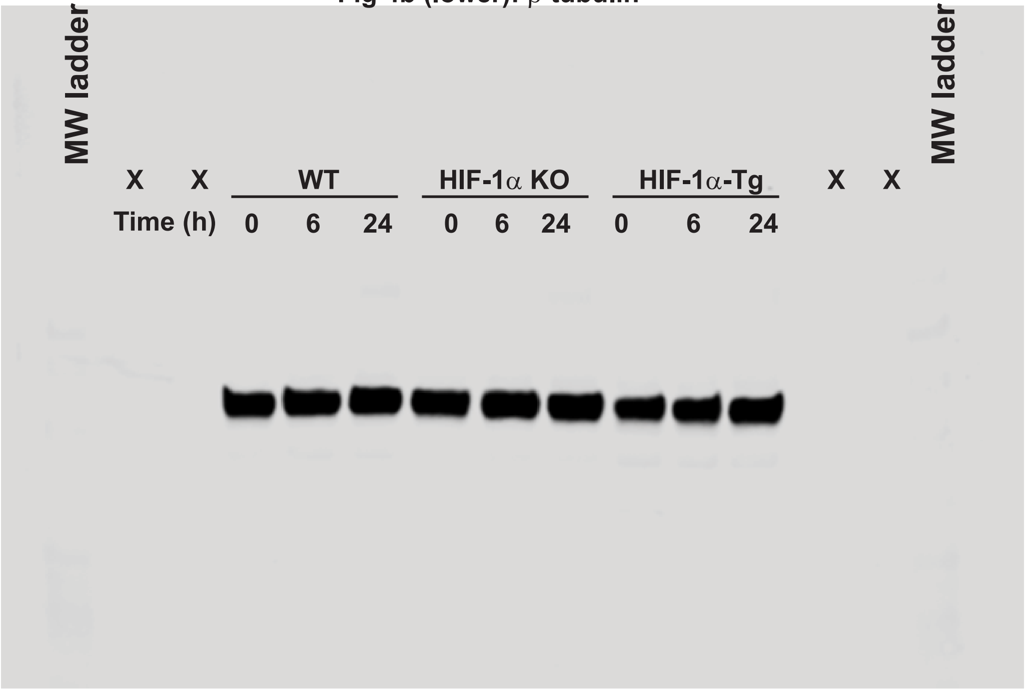

Both images shown above were captured using the Licor Odyssey CLx imaging system with Image Studio software.
